# *Colletotrichum* Species Associated with Anthracnose Disease of Watermelon (*Citrullus lanatus*) in China

**DOI:** 10.3390/jof8080790

**Published:** 2022-07-28

**Authors:** Zhen Guo, Chao-Xi Luo, Hui-Jie Wu, Bin Peng, Bao-Shan Kang, Li-Ming Liu, Meng Zhang, Qin-Sheng Gu

**Affiliations:** 1 Key Laboratory of Fruit and Cucurbit Biology, Zhengzhou Fruit Research Institute, Chinese Academy of Agricultural Sciences, Zhengzhou 450009, China; guozhenguorui@163.com (Z.G.); wuhuijie@caas.cn (H.-J.W.); pengbin@caas.cn (B.P.); kangbaoshan@caas.cn (B.-S.K.); liuliming@caas.cn (L.-M.L.); 2 Key Laboratory of Horticultural Plant Biology, Ministry of Education, College of Plant Science and Technology, Huazhong Agricultural University, Wuhan 430070, China; cxluo@mail.hzau.edu.cn; 3 Department of Plant Pathology, Henan Agricultural University, Zhengzhou 450002, China; zm2006@126.com

**Keywords:** multi-locus phylogeny, pathogenicity, plant pathogen, taxonomy

## Abstract

*Colletotrichum* species are important plant pathogens, causing anthracnose in virtually every crop grown throughout the world. However, little is known about the species that infect watermelon. A total of 526 strains were isolated from diseased watermelon samples of eight major watermelon growing provinces in China. Phylogenetic analyses using seven loci (ITS, *gadph*, *chs-1*, *his3*, *act*, *tub2*, and *gs*) coupled with morphology of 146 representative isolates showed that they belonged to 12 known species of *Colletotrichum*, including *C. aenigma*, *C. chlorophyti*, *C. fructicola*, *C. jiangxiense*, *C. karstii*, *C. magnum*, *C. nymphaeae*, *C. nigrum*, *C. orbiculare*, *C. plurivorum*, *C. sojae*, and *C. truncatum* and three new species, here described as *C. citrulli*, *C. kaifengense*, and *C. qilinense*. *Colletotrichum orbiculare* was the dominant species. Pathogenicity tests revealed that all isolates of the species described above were pathogenic, with *C. magnum* and *C. kaifengense* being the most aggressive to leaves and fruits, respectively. This is the first report of *C. aenigma*, *C. chlorophyti*, *C. fructicola*, *C. jiangxiense*, *C. nymphaeae*, *C. nigrum*, *C. plurivorum*, and *C. sojae* on watermelon. These findings shed light on the *Colletotrichum* spp. involved in watermelon anthracnose and provide useful information for implementing effective control of watermelon anthracnose in China.

## 1. Introduction

*Colletotrichum* is the most common and important genus of plant pathogenic fungi, saprobes, and endophytes [1,2,3]. Species of *Colletotrichum* spp. infect numerous plant crops worldwide, e.g., apple (*Malus pumila*), chili (*Capsicum* spp.), coffee (*Coffea* spp.), grape (*Vitis vinifera*), longan (*Dimocarpus longan*), mango (*Mangifera indica*), olive (*Canarium album*), orange (*Citrus* spp.), pear (*Pyrus* spp.), peach (*Prunus persica*), strawberry (*Fragaria ananassa*), and tea (*Camellia* spp.) [4,5,6,7,8,9,10,11,12,13,14,15,16,17,18,19,20,21].

During the first half of the 20th century, many species of the plant-pathogenic fungal genus *Colletotrichum* were defined, relying on the hosts from which they were originally isolated [22]. Based on his revision of the genus primarily on morphological characteristics in culture, Von Arx reduced approximately 750 species to only 11 [23]. However, identifying *Colletotrichum* species is challenging due to the instability of their morphological characteristics, which are affected by experimental methods and conditions [1,24,25]. Along with the increased availability of DNA sequencing technology, a very large volume of DNA sequence data has been generated, allowing fungal taxonomy through phylogenetics, including genealogical concordance [26,27]. Following the adoption of multi-locus phylogenetic analysis together with morphological characteristics, the classification and species concepts in *Colletotrichum* taxa worldwide have changed significantly [1,28,29,30,31,32,33]. Nearly all acknowledged species studied were grouped into 16 *Colletotrichum* species complexes, and more than 16 singleton species have been identified [9,30,31,32,33,34,35,36,37,38,39,40,41,42,43].

Watermelon (*Citrullus lanatus* (Thunb.) Matsum. et Nakai), belongs to the xerophytic genus *Citrullus* Schrad. ex Eckl. et Zeyh of the botanical family *Cucurbitaceae* and is one of the most important commercial crops worldwide [44]. It is the third-most widespread fruit crop after apple and orange in China (China Agriculture Research System Statistical Data 2018). In 2019, the total area harvested in China was 1,471,581 ha, with a yield of 60,861,241 t, accounting for 60.6% of the world’s production of watermelon [45]. However, anthracnose diseases are one of the important factors, limiting the commercial production of watermelon [26]. Anthracnose displays spots and blights on the aerial parts of plant in the fields. Owing to anthracnose, watermelon production can be reduced by 5–20%, and even causes no harvest [46]. Moreover, it is an important post-harvest pathogen and becomes active after the fruit has been stored or appears on the market shelf. Owing to *Colletotrichum* disease, up to 100% of the fruit can be lost during storage and transportation [2].

*Cucurbitaceae* crops, especially watermelon (*Citrullus lanatus*), melon (*Cucumis melo*), and cucumber (*Cucumis sativus*), are important plant hosts of *Colletotrichum* spp. [47]. Seven *Colletotrichum* species have been reported in watermelon, belonging to *C. gloeosporioides*, *C. gloeosporioides* f.sp. cucurbitae, *C. karstii*, *C. magnum*, *C. scovillei*, *C. orbiculare*, and *C. truncatum* [32,37,38,48,49,50,51]. First described from cucumber, the species *C. gloeosporioides* f.sp. cucurbitae is widely regarded as a synonym for *C. orbiculare* [52]. However, *Colletotrichum* species associated with watermelon remain largely unresolved, with only six individual species identified. Moreover, these studies used a limited number of samples and areas, therefore species diversity might have been underestimated.

The objectives of the present study were as follows: (i) to identify the *Colletotrichum* species causing anthracnose in watermelon in the major production provinces in China based on multi-locus phylogenetic analyses combined with morphological characterization; (ii) to evaluate the pathogenicity of the different *Colletotrichum* species.

## 2. Materials and Methods

### 2.1. Collection and Isolates

**Figure 1:**
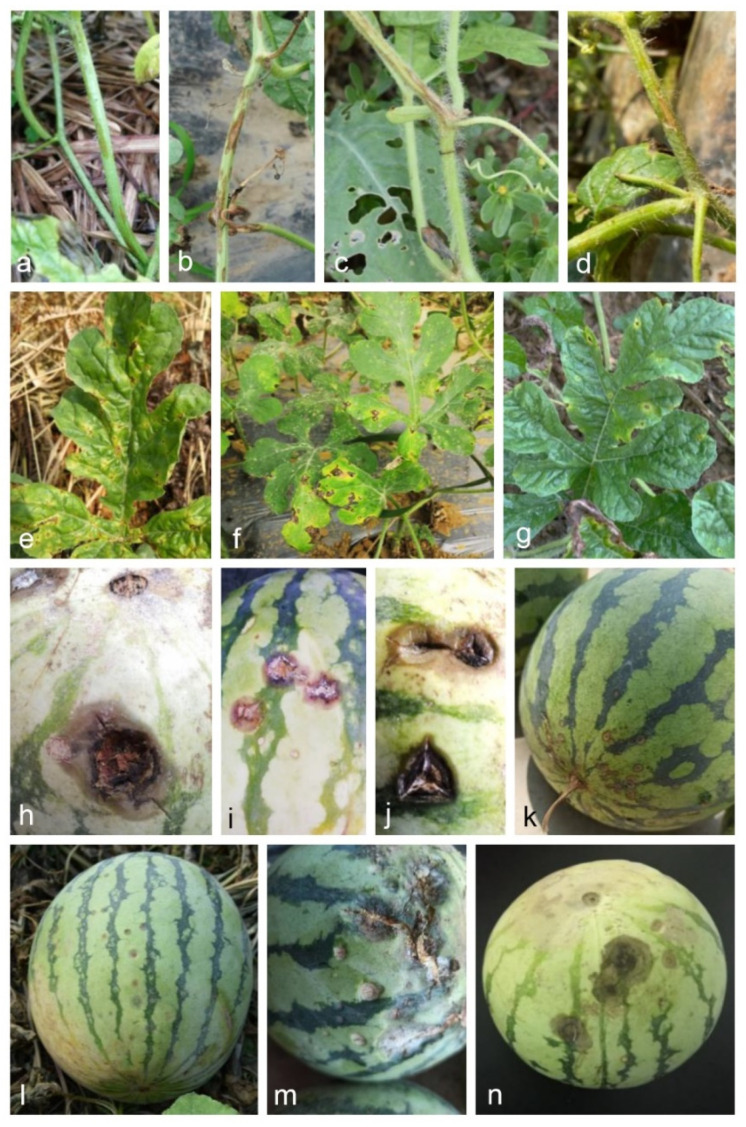
Symptoms of watermelon anthracnose on leaves, stems, and fruits in the field. (**a**–**d**) Symptoms on leaves of *Citrullus lanatus*; (**e**–**g**) symptoms on stems of *Citrullus lanatus*; (**h**–**n**) symptoms on fruits of *Citrullus lanatus*.

**Figure 2:**
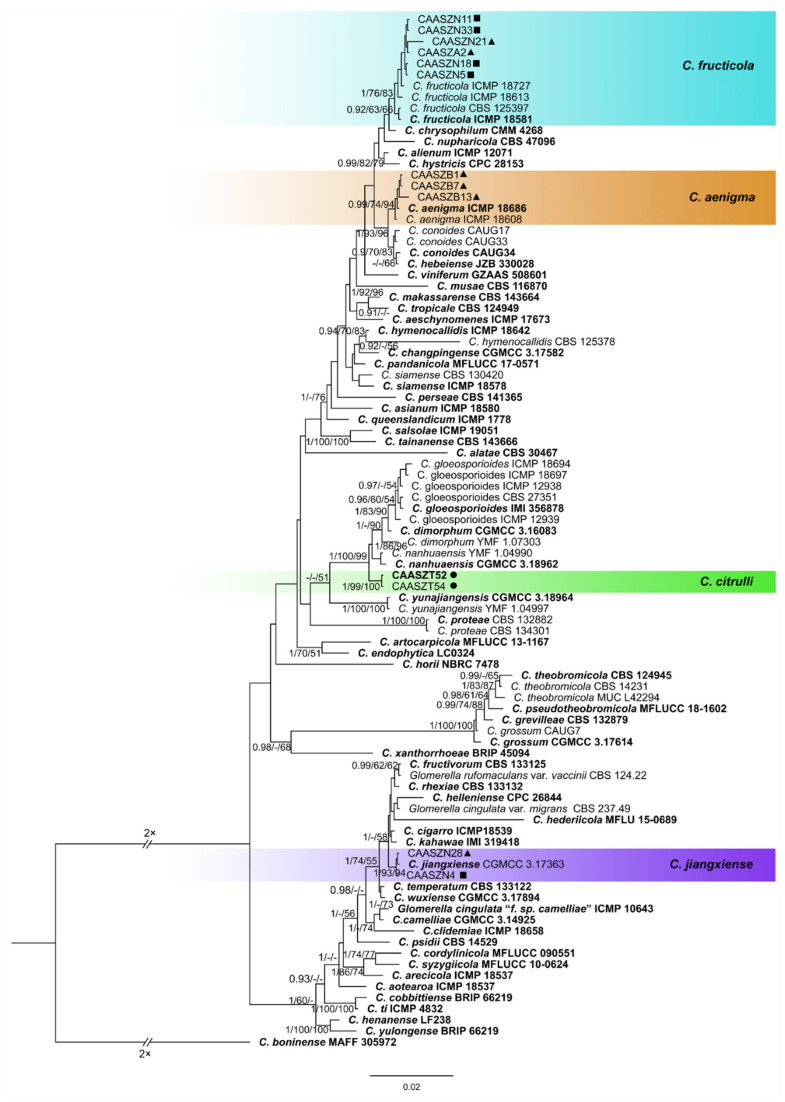
A Bayesian inference phylogenetic tree of 92 isolates in the *C. gloeosporioides* species complex. The species *C. boninense* (MAFF 305972) was selected as an outgroup. The tree was built using concatenated sequences of the ITS, *gadph*, *chs-1*, *his3*, *act*, and *tub2* genes. Bayesian posterior probability (PP ≥ 0.90), MP bootstrap support values (MP ≥ 60%), and iqtree bootstrap support values (ML ≥ 50%) were shown at the nodes (PP/MP/ML). Ex-type isolates are in bold. Colored blocks indicate clades containing isolates from *Citrullus lanatus* in this study; triangles indicate strains isolated from leaves, rectangle indicate strains isolated from stems, circles indicate strains isolated from fruits. The scale bar indicates 0.02 expected changes per site.

The survey was conducted from 2018 to 2020 in watermelon fields in various geographical areas of China. Samples were collected from eight provinces (Henan, Jiangsu, Zhejiang, Jilin, Liaoning, Hebei, Jiangxi, and Hainan) (Table 1). Anthracnose symptoms on watermelon leaves were small circular or irregular spots, pale brown in the center with medium to dark brown margins, while rows of fusiform brown sunken spots were formed on stems (Figure 1a–g). Three symptom types were observed on fruits: (1) small round sunken spots (Figure 1k,l) and (2) large brown or black sunken rot lesions, forming orange conidia under humid conditions (Figure 1h–j,m,n).

Samples were surface-sterilized by dipping in 75% ethanol for 10 s and rinsing three times with sterile distilled water. Tissue pieces (5 mm) were excised from areas neighboring the diseased tissue. Excised tissue was placed onto potato dextrose agar (PDA, 20% diced potato, 2% dextrose, and 1.5% agar, and distilled water) after surface sterilization (70% ethanol for 15 s, 5% NaOCl for 5 min, washed three times in sterile water and dried on sterilized filter paper) [53]. The plates were incubated at 27 °C in the dark for 5 d. Six single colonies of each strain were isolated using the single-spore isolation method [54]. All isolates were stored in 25% glycerol at −80 °C. Type specimens of the new species from this study were deposited in the Mycological Herbarium, Institute of Microbiology, Chinese Academy of Sciences, Beijing, China (HMAS), and ex-type living cultures were deposited in the China General Microbiological Culture Collection Center (CGMCC), Beijing, China.

### 2.2. DNA Extraction, PCR Amplification, and Sequencing

Isolates were transferred to fresh PDA plates and incubated at 27 °C for 7–19 days. Genomic DNA of 146 representative isolates was extracted using the Ezup Column Fungi Genomic DNA Purification Kit (Sangon Biotech, Shanghai, China). Seven loci, including the internal transcribed spacer regions and intervening 5.8S nrRNA gene (ITS) and partial sequences of the glyceraldehyde-3-phosphate dehydrogenase (*gadph*), chitin synthase 1 (*chs-1*), actin (*act*), histone3 (*his3*), beta-tubulin (*tub2*), and glutamine synthetase (*gs*) genes were amplified and sequenced using the primer pairs ITS-1F [55] + ITS-4 [56], GDF1 + GDR1 [57], CHS-354R + CHS-79F [58], ACT-512 F + ACT-783R [58], CYLH3F + CYLH3R [59], T1 [60] +Bt2b [61] and GSF1 + GSR1 [57], respectively. PCR was performed in a total volume of 25 μL. The PCR mixture contained 1 μL 20× diluted genomic DNA, 12.5 μL 2 × Rapid Taq Master Mix (Vazyme, Nanjing, China), and 0.2 μM of each primer. The PCR reactions were mostly as described by Woudenberg et al. (2009) [62], but were modified by using an annealing temperature of 58 °C for *act*, *gadph*, and *chs-1*, 56 °C for *tub2*, *his3*, and *gs*. PCR amplifications were performed in Mastercycler X50 (Eppendorf, Hamburg, Germany). PCR amplicons were purified and sequenced at the Sangon Biotech (Shanghai, China) Company, Ltd. Forward and reverse primers were assembled to obtain consensus sequences using DNAMAN (v. 9.0; Lynnon Biosoft, San Ramon, USA). Sequences generated in this study were deposited in GenBank (Supplementary Table S1).

### 2.3. Phylogenetic Analyses

The DNA sequences were aligned using MAFFT v. 7 [63]. and manually adjusted using MEGA (version 11.0.10, Mega Limited, Auckland, New Zealand) where necessary. Multiple sequences of ITS, *gadph*, *act*, *tub2*, *chs-1*, and *gs* were concatenated using SequenceMatrix 1.8 [64]. A Markov Chain Monte Carlo (MCMC) algorithm was used to generate phylogenetic trees with Bayesian inference (BI) using MrBayes v. 3.2.6 [65] for the combined sequence datasets. Best-fit models of nucleotide substitution for each gene partition were determined by Akaike information criterion (AIC) of MrModeltest v. 2.3 [66] (Table 2). Two analyses of four MCMC chains were conducted from random trees with 6 × 10^6^ generations for *C. orbiculare* and *C. gloeosporioides* species complexes, and 2 × 10^6^ generations for the *C. boninense*, *C. orchidearum*, *C. magnum* species complexes, and *C. acutatum*, *C. truncatum* species complexes and the related reference species involved in the same phylogenetic tree. The analyses were sampled every 1000 generations.

Alignment gaps were treated as missing and data all characters were unordered and of equal weight. The first 25% of trees were discarded as the burn-in phase of each analysis and posterior probabilities were determined from the remaining trees. Convergence of all parameters (i.e., effective sample sizes >200) was visually confirmed using Tracer 1.7.1 [67]. The maximum parsimony analyses (MP) were performed on the multi-locus alignment using PAUP (Phylogenetic Analysis Using Parsimony) v. 4.0a169 [68]. Phylogenetic trees were generated using the heuristic search option with 1000 random sequence additions and tree bisection and reconstruction (TBR) as the branch-swapping algorithm. Maxtrees were set up to 5000, branches of zero length were collapsed, and all multiple parsimonious trees were saved. Clade stability was assessed by bootstrap analysis with 1000 replicates. Afterward, tree length (TL), consistency index (CI), retention index (RI), rescaled consistency index (RC), and homoplasy index (HI) were calculated for the resulting tree. Furthermore, maximum likelihood (ML) analyses were implemented on the multi-locus alignments using the IQ-TREE 2 [69]. Clade stability was assessed using a bootstrap analysis with 1000 replicates. Phylogenetic trees were visualized in FigTree v. 1.4.4 [70]. The alignments and phylogenetic trees were deposited in TreeBASE (submission number: 29157).

The phylogenetically related ambiguous species were analyzed using the Genealogical Concordance Phylogenetic Species Recognition (GCPSR) model with a pairwise homoplasy index (PHI) test as described by Quaedvlieg et al. (2014) [26]. The PHI test was performed in SplitsTree4 [71,72,73] to determine the recombination level within phylogenetically closely related species using a six-locus concatenated dataset (ITS, *gadph*, *chs-1*, *his3*, *act,* and *tub2*). Pairwise homoplasy index results below 0.05 indicated significant recombination in the dataset. The relationship between closely related species was visualized by constructing a splits graph.

### 2.4. Morphological Analysis

Mycelial plugs (5-mm diam) from the margin of actively growing cultures were transferred on PDA, oatmeal agar (OA) [53], and synthetic nutrient-poor agar medium (SNA) [74] and incubated at 27 °C in the dark. Colony characteristics were noted after 9 d, and colony diameters were measured daily for 3 d, 5 d, and 7 d to calculate their mycelial average growth rates (mm/d). Additionally, the shape, color, and size of acervali, conidia, conidiophores, asci, ascospores, and seta were observed using light microscopy (Nikon SMZ-1500 and Olympus BX51, Japan or Leica TCS SP5, Germany). Moreover, to determine their sizes, 30 conidia, seta, or ascospores were measured. The formation of conidial appressoria was induced by dropping a conidial suspension in 1% glucose (10^7^ conidia/mL; 50 μL) on a concavity slide, placed inside plates containing moistened filter papers with sterile distilled water, and then incubated at 27 °C in darkness. After 48 h, the sizes of 30 conidial appressoria were measured.

### 2.5. Prevalence

To determine the prevalence of *Colletotrichum* species in the sampled provinces, *Colletotrichum* species were isolated from infected watermelon organs (leafs, stems, or fruits). The isolation rate (IR) for each species was calculated using the formula, IR% = (Ns/Nt) × 100, where Ns is the number of isolates from the same species and Nt is the total number of isolates from each sample-collected province [11,14].

### 2.6. Pathogenicity Tests

Sixteen representative isolates of each *Colletotrichum* species were used in the pathogenicity tests on detached leaves of *Citrullus lanatus* cv. Hongheping (5–6 true leaves). Healthy leaves were collected from plants growing in pots in a greenhouse. The leaves were washed with tap water, then submerged in 75% ethanol for 30 s, washed three times with sterile water, and finally air-dried on sterilized filter paper. The leaves were inoculated using the wound/drop and non-wound/drop inoculation methods [75,76]. A drop of 10 µL spore suspension (10^6^ conidia/mL) was individually placed onto the left side of the leaf after wounding once by pin-pricking the upper surface with a sterilized needle (insect pin, 0.5 mm diam). A 10 µL drop of sterile water was placed on the right side of the same leaf as a control. The process was repeated with unwounded leaves. The leaves were incubated at 27 °C and 100% humidity in the dark. The infection rate was calculated 4 d post-inoculation (dpi) using the formula PI (%) = Nf/Np × 100, where Nf is the number of infected points and Np is the total number of inoculated points [19]. All isolates were tested in triplicate.

Pathogenicity tests were also performed on detached mature watermelon fruits of *Citrullus lanatus* cv. Motong in triplicate. Healthy-looking fruits were collected from plants growing in the greenhouse. The fruits were washed with tap water, then submerged in 75% ethanol for 30 s and washed three times with sterile water, finally air-dried on sterilized filter paper. Wound/drop and non-wound/drop inoculation methods were used [28,75,76]. For the wound inoculation, a drop of 10 µL spore suspension (10^6^ conidia/mL) was placed directly onto fruit surfaces after wounding once by pin-pricking with a sterilized needle (5 mm deep). In the non-wound inoculation, the same spore suspension was placed directly on the unwounded watermelon fruit skin. Sterile water was used as the control. The fruits were incubated at 27 °C and at 100% humidity in the dark. The infection rate was calculated at 6 dpi using the formula PI (%) = Nf/Np × 100, where Nf is the number of infected points and Np is the total number of inoculated points [19].

## 3. Results

### 3.1. Collection of Watermelon Anthracnose Samples and Strain Isolation

Watermelon anthracnose was common in all the fields and provinces surveyed. A total of 224 diseased watermelon samples (146 leaves, 57 stems, and 21 fruits) were collected from eight provinces in China. From them, 526 *Colletotrichum* strains were isolated based on the colony morphologies on PDA and ITS sequence data (Table 1). Based on megablast searches in GenBank using ITS sequences, the colony morphological characteristics on PDA, and morphological characteristics conidia isolates were primarily divided into eight groups. Of those, 146 representative isolates were selected for further analysis.

### 3.2. Multi-Locus Phylogenetic Analyses

**Figure 3:**
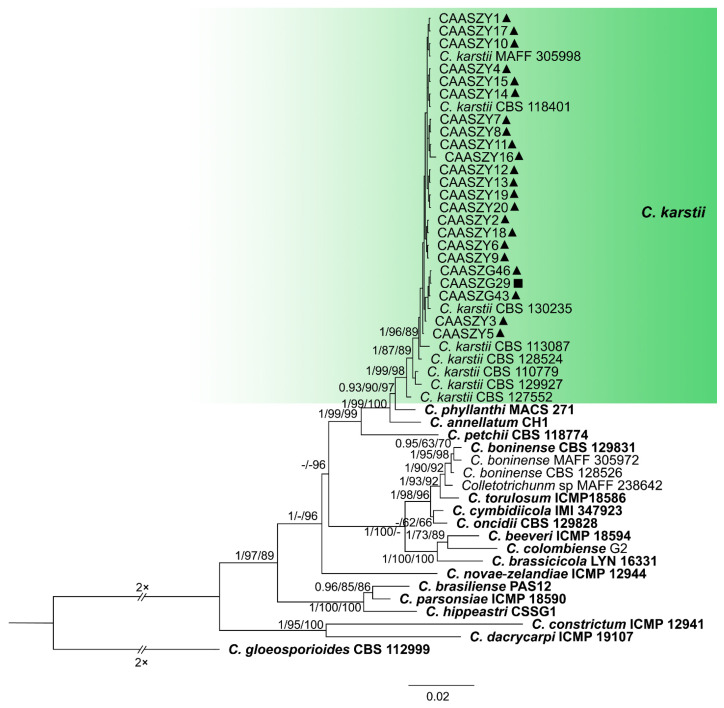
A Bayesian inference phylogenetic tree of 50 isolates in the *C. boninense* species complex. The species *C. gloeosporioides* (CBS 112999) was selected as an outgroup. The tree was built using concatenated sequences of the ITS, *gadph*, *chs-1*, *his3*, *act*, and *tub2* genes. Bayesian posterior probability (PP ≥ 0.90), MP bootstrap support values (MP ≥ 60%), and iqtree bootstrap support values (ML ≥ 50%) were shown at the nodes (PP/MP/ML). Ex-type isolates are in bold. Colored blocks indicate clades containing isolates from *Citrullus lanatus* in this study; triangles indicate strains isolated from leaves, rectangle indicate strains isolated from stems. The scale bar indicates 0.02 expected changes per site.

**Figure 4:**
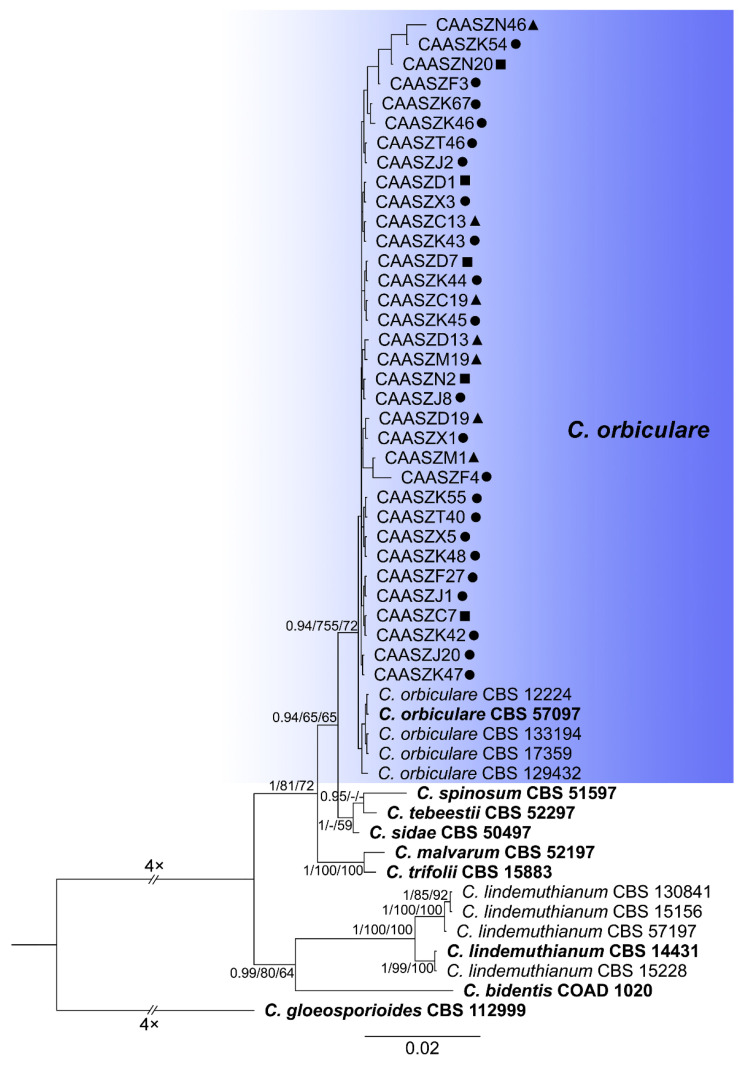
A Bayesian inference phylogenetic tree of 50 isolates in the *C. orbiculare* species complex. The species *C. gloeosporioides* (CBS 112999) was selected as an outgroup. The tree was built using concatenated sequences of the ITS, *gadph*, *chs-1*, *his3*, *act*, *tub2*, and *GS* genes. Bayesian posterior probability (PP ≥ 0.90), MP bootstrap support values (MP ≥ 60%), and iqtree bootstrap support values (ML ≥ 50%) were shown at the nodes (PP/MP/ML). Ex-type isolates are in bold. Colored blocks indicate clades containing isolates from *Citrullus lanatus* in this study; triangles indicate strains isolated from leaves, rectangle indicate strains isolated from stems, circles indicate strains isolated from fruits. The scale bar indicates 0.02 expected changes per site.

**Figure 5:**
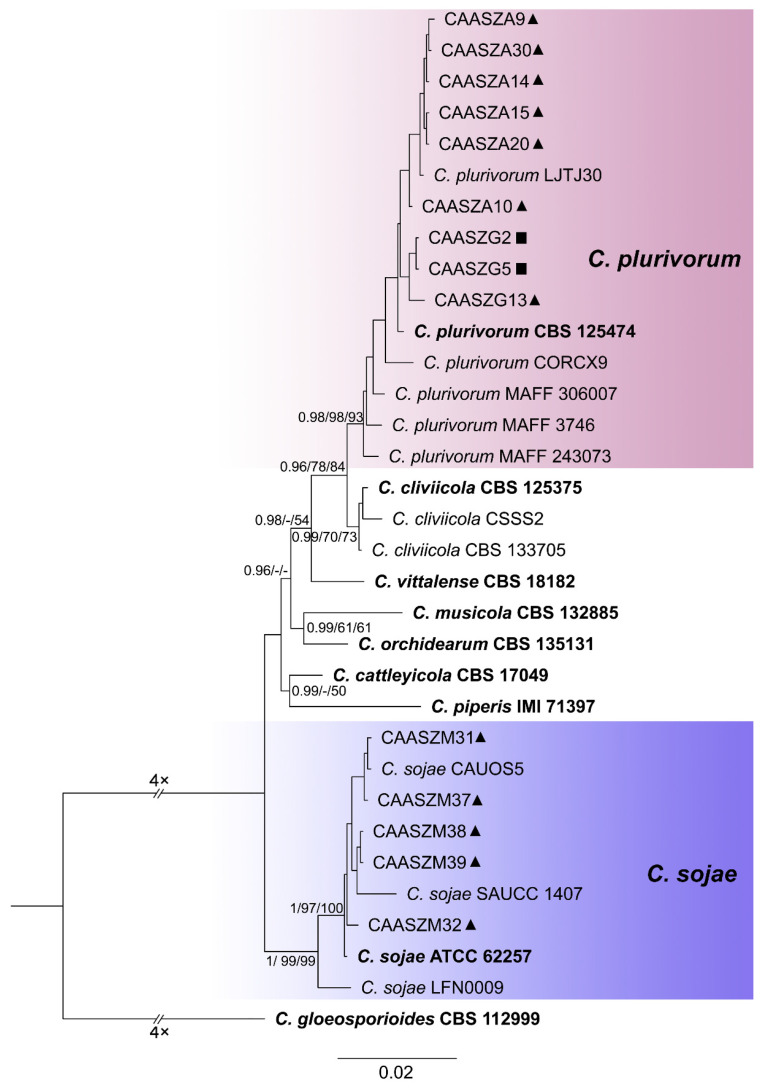
A Bayesian inference phylogenetic tree of 32 isolates in the *C. orchidearum* species complex. The species *C. gloeosporioides* (CBS 112999) was selected as an outgroup. The tree was built using concatenated sequences of the ITS, *gadph*, *chs-1*, *his3*, *act*, and *tub2* genes. Bayesian posterior probability (PP ≥ 0.90), MP bootstrap support values (MP ≥ 60%), and iqtree bootstrap support values (ML ≥ 50%) were shown at the nodes (PP/MP/ML). Ex-type isolates are in bold. Colored blocks indicate clades containing isolates from *Citrullus lanatus* in this study; triangles indicate strains isolated from leaves, rectangle indicate strains isolated from stems. The scale bar indicates 0.02 expected changes per site.

**Figure 6:**
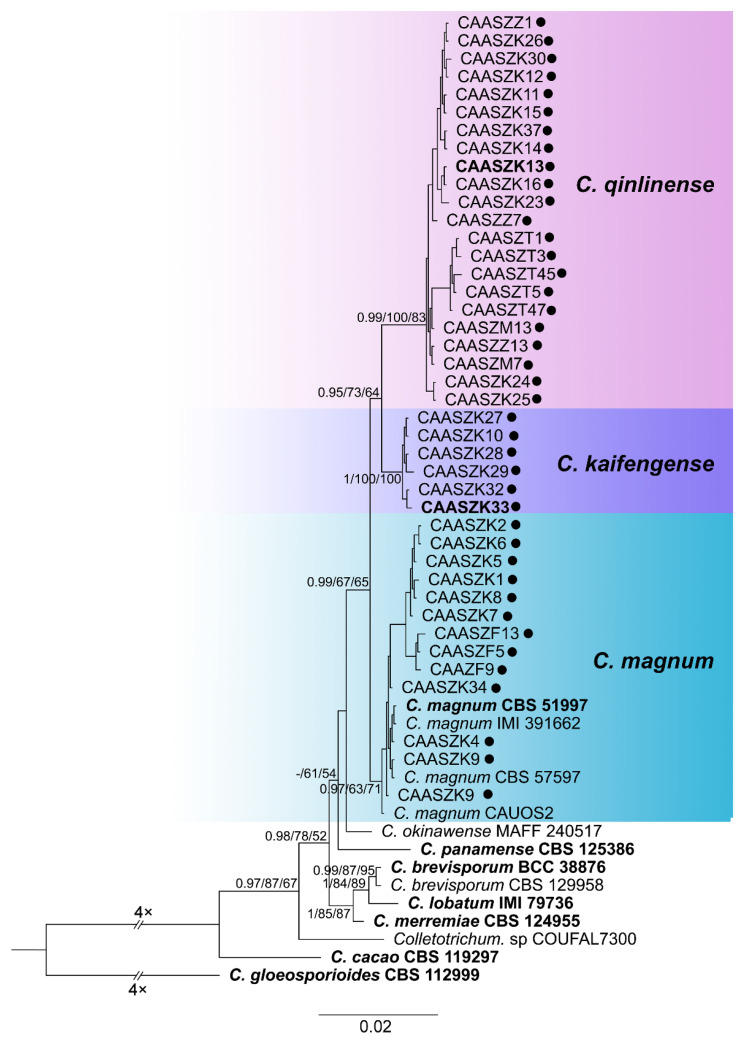
A Bayesian inference phylogenetic tree of 52 isolates in the *C. magnum* species complex. The species *C. gloeosporioides* (CBS 112999) was selected as an outgroup. The tree was built using concatenated sequences of the ITS, *gadph*, *chs-1*, *his3*, *act*, and *tub2* genes. Bayesian posterior probability (PP ≥ 0.90), MP bootstrap support values (MP ≥ 60%), and iqtree bootstrap support values (ML ≥ 50%) were shown at the nodes (PP/MP/ML). Ex-type isolates are in bold. Colored blocks indicate clades containing isolates from *Citrullus lanatus* in this study; circles indicate strains isolated from fruits. The scale bar indicates 0.02 expected changes per site.

**Figure 7:**
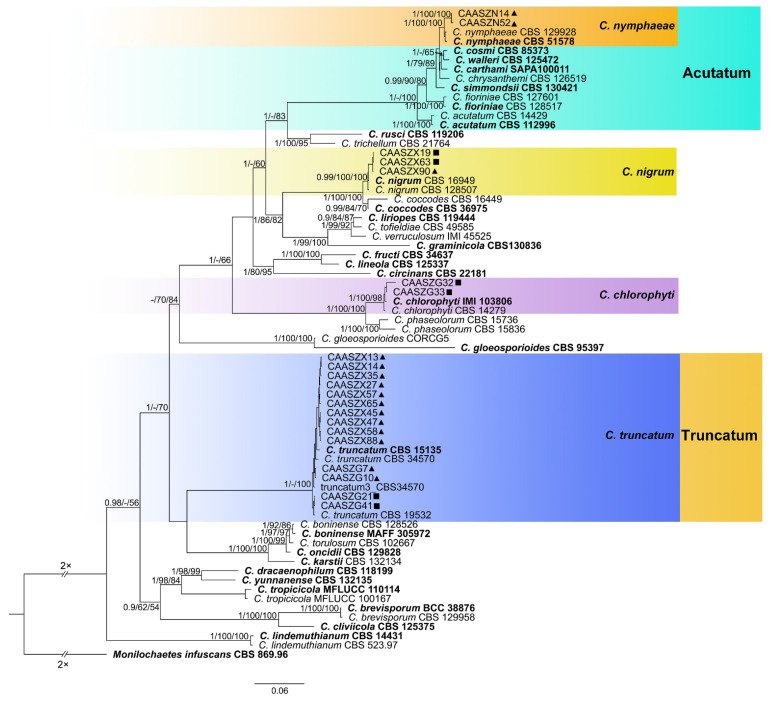
Phylogenetic tree generated by Bayesian inference based on concatenated sequences of the ITS, *gadph*, *chs-1*, *his3*, *act* and *tub2* genes. *Monilochaetes infuscans* (CBS 869.96) was selected as an outgroup. Bayesian posterior probability (PP ≥ 0.90), MP bootstrap support values (MP ≥ 60%), and iqtree bootstrap support values (ML ≥ 50%) were shown at the nodes (PP/MP/ML). Ex-type isolates are in bold. Colored blocks indicate clades containing isolates from *Citrullus lanatus* in this study; triangles indicate strains isolated from leaves, rectangle indicate strains isolated from stems. The scale bar indicates 0.06 expected changes per site.

The 146 representative isolates and related *Colletotrichum* species including the outgroup were subjected to multi-locus phylogenetic analyses (Supplementary Tables S1 and S2) with 7-locus phylogenetic analyses (ITS, *gadph*, *chs-1*, *his3*, *act, tub2* and *gs*) for *C. orbiculare* species complexes, 6-locus phylogenetic analyses (ITS, *gadph, chs-1, his3, act, and tub2*) for those belonging to the *C. gloeosporioides, C. boninense*, *C. magnum,* and *C. orchidearum* species complexes, or with 5-locus phylogenetic analyses (ITS, *gadph*, *chs-1*, *his3*, *act,* and *tub2*) for other species complexes of without available *his3* sequences. The consensus tree obtained from maximum parsimony and maximum likelihood analyses confirmed the tree topology obtained with Bayesian inference (Figure 2, Figure 3, Figure 4, Figure 5, Figure 6 and Figure 7).

**Figure 8:**
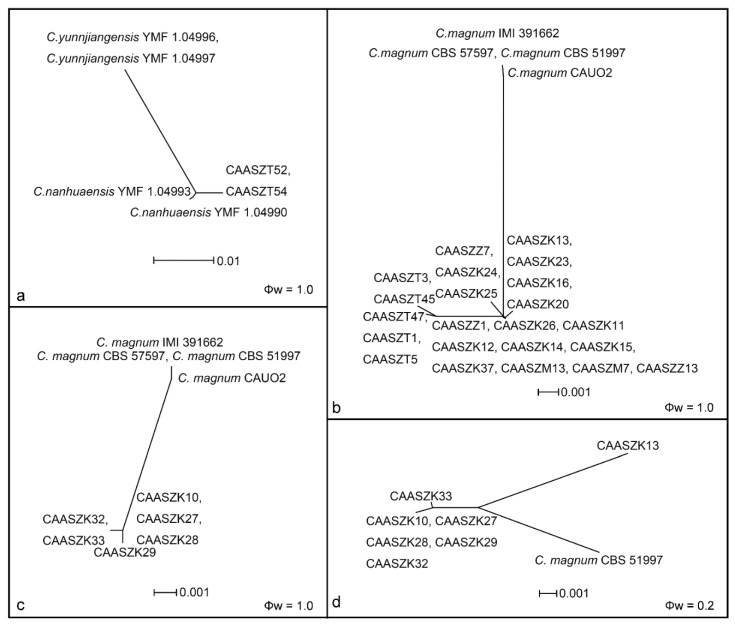
The result of the pairwise homoplasy index (PHI) tests of closely related species using both LogDet transformation and Splits decomposition. The PHI of *C. citrulli* (**a**) or *C. kaifengense* (**b**) or *C. qilinense* (**c**) or *C. kaifengense* and *C. qilinense* (**d**) and their phylogenetically related isolates or species, respectively. PHI test value (Φw) < 0.05 indicate significant recombination within the dataset.

The phylogenetic tree was constructed for the isolates of the *C. gloeosporioides* species complex, in which six isolates were clustered with *C. fructicola*, six with *C. aenigma*, and two with *C. jiangxiense*. In addition, the two isolates formed a distinct clade (Bayesian posterior probabilities value 1/PAUP bootstrap support value 99/iqtree bootstrap support value 100) as a sister group to *C. nanhuaensis*, which clustered distantly from any known species in the complex (Figure 2). For isolates in the *C. boninense* species complex, 23 isolates were clustered with *C. karstii* (Figure 3). For the isolates in the *C. orbiculare* species complex, 34 isolates were clustered with *C. orbiculare* (Figure 4). For isolates of *C. orchidearum* species complex, 14 isolates were clustered in two clades, 9 with *C. plurivorum*, and 5 with *C. sojae* (Figure 5). In the *C. magnum* species complex, 14 isolates were clustered with *C. magnum*. The remaining 29 isolates were clustered in two distinct clades (1/99/100, 1/99/96) as sisters to *C. magnum*, and distanted from any known species in the complex (Figure 6). The remaining 21 isolates were clustered in four clades corresponding to *C. nymphaeae* (2 isolates), *C. nigrum* (3 isolates), *C.* *chlorophyti* (2 isolates), and *C. truncatum* (14 isolates) (Figure 7). The PHI test indicated no significant recombination events between *C. citrulli* and *C.nanhuaensis* (Figure 8a). In addition, the results showed that there were no significant recombination events between *C. qilinense* and *C. magnum* (Figure 8b), or between *C. kaifengense* and *C. magnum* (Figure 8c). The results revealed no significant recombination events between *C. kaifengense*, a sister group of *C. qilinense*, and *C. magnum* (Figure 8b,c).

### 3.3. Multi-Locus Phylogenetic Analyses

**Figure 9:**
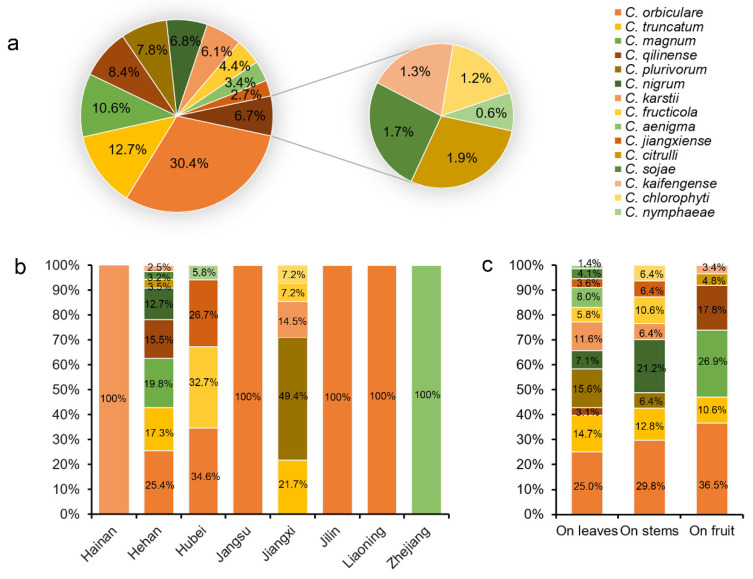
The prevalence of *Colletotrichum* species isolated from watermelon. (**a**) Overall isolation rate (%) of *Colletotrichum* species; (**b**,**c**) isolation rate (%) of *Colletotrichum* species from part of sampled province (**b**) and watermelon organs (**c**), respectively.

Analyses of the prevalence of the *Colletotrichum* species associated with watermelon in China showed that *C. orbiculare* isolates (160 isolates, 28.9% of the total isolates) were predominantly isolated from five provinces (Henan, Hubei, Liaoning, Jilin, and Jiangsu), followed by *C. truncatum* (67 isolates, 8.8%, isolated from Henan and Jiangxi), *C. qilinense* (44 isolates, 8.4%, isolated from Henan), *C. plurivorum* (41 isolates, 8.4%, isolated from Jiangxi), *C. magnum* (56 isolates, 8.4%, isolated from Henan), *C. nigrum* (37 isolates, 6.8%, isolated from Henan), *C. karstii* (32 isolates, 6.1%, isolated from Jiangxi and Hainan), *C. fructicola* (23 isolates, 4.4%, isolated from Jiangxi and Hubei), and *C. aenigma* (18 isolates, 3.4%, isolated from Zhejiang). The remaining six species account for 9.3% of total isolates (Figure 9a). These results suggested that *C. orbiculare* is the most prevalent species of *Colletotrichum* sp. on watermelon in China (Figure 9a).

Analyses of the isolation rate of 15 *Colletotrichum* species in each sampled province revealed that *C. orbiculare* was commonly isolated in Henan and Hubei provinces, accounting for 25.4% and 34.6% of the obtained isolates, respectively (Figure 9b). In the Jiangxi province, *C. plurivorum* strains were dominant, accounting for 49.4% of total isolates from this province (Figure 9b). Moreover, all the *C. orbiculare* isolates were from Liaoning, Jilin, and Jiangsu provinces, and *C. aenigma* isolates were isolated from Zhejiang province (Figure 9b). Analyses of the *Colletotrichum* species isolated from three organs of watermelon revealed that 11 *Colletotrichum* species (*C. orbiculare*, *C. truncatum*, *C. qilinense*, *C. plurivorum*, *C. nigrum*, *C. karstii*, *C. fructicola*, *C. aenigma*, *C. jiangxiense*, *C. sojae,* and *C. nymphaeae*) were isolated from leaves, 8 (*C. orbiculare*, *C. karstii*, *C. truncatum*, *C. plurivorum*, *C. fructicola*, *C. nigrum, C. jiangxiense*, and *C. chlorophyti*) were isolated from stems, and 5 (*C. orbiculare*, *C. citrulli*, *C. magnum*, *C. qilinnense,* and *C. kaifengense*) were isolated from the fruits. Of those, *C. orbiculare* strains were dominant and accounted for 25.0%, 29.8%, and 36.5% of total isolates from leaves, stems, and fruits, respectively (Figure 9c).

### 3.4. Pathogenicity Assay

**Figure 10:**
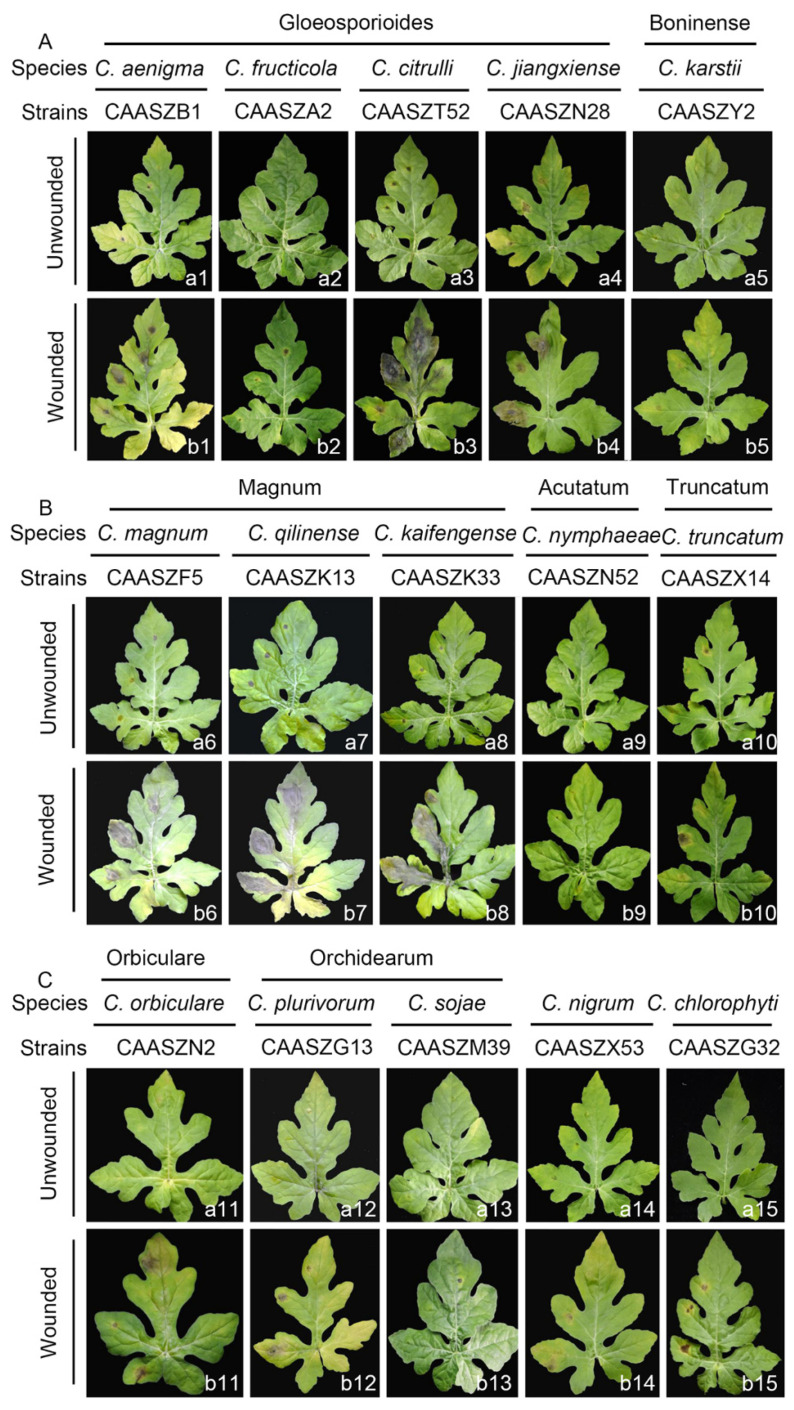
Symptoms of watermelon leaves (*Citrullus lanatus* cv. Hongheping) induced by inoculation of spore suspensions of 15 *Colletotrichum* spp. under unwounded and wounded conditions. The symptoms caused by these species were photographed at 4 dpi. (**a1**–**a15**) Under wounded condi-tions, symptoms induced by the isolates/species belonging to the *C. gloeosporioides, C. boninense, C. magnum, C. acutatum, C. truncatum, C. orbiculare*, and *C. orchidearum* complexe species or singleton species, respectively. (**b1**–**b15**) Under unwounded conditions, symptoms induced by the iso-lates/species belonging to the *C. gloeosporioides, C. boninense, C. magnum, C. acutatum, C. truncatum, C. orbiculare, C. orchidearum* complexe species or singleton species, respectively. The symptoms induced by the isolates/species belonging to the *C. gloeosporioides* complex and the *C. boninense* complex (**A**), the *C. magnum* complex (**B**), and other complexes or singleton species (**C**), respectively. The inoculation was conducted by dropping 1 × 10^7^ spores (conidia or ascospores) per mL on about 6–8 detached true leaves of *Citrullus lanatus* cv. Hongheping in three replicates after wounded by pin-pricking each leaf for one time with a sterilized needle (wounded) or kept unwounded (unwounded).

**Figure 11:**
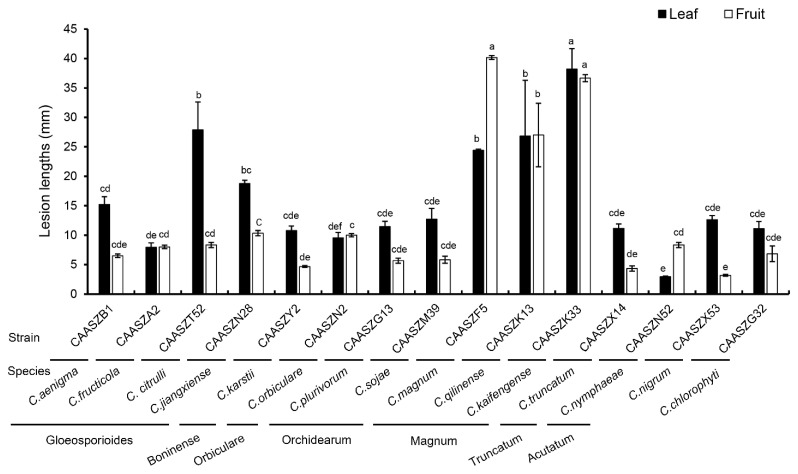
Lesion lengths on wounded watermelon leaves (*Citrullus. lanatus* cv. Hongheping) and fruits (*Citrullus. lanatus* cv. Motong) at 4 dpi and 6 dpi, respectively, induced by conidial suspensions of 16 representative isolates of 16 *Colletotrichum* spp. The involved isolates and their belonging are indicated at the bottom of the bars. Data were analyzed with SPSS Statistics 19.0 (WinWrap Basic; http://www.winwrap.com (accessed on 18 December 2021)) by one-way analysis of variance, and means were compared using Duncan’s test at a significance level of *p* = 0.05. Letters over the error bars indicate the significant difference at the *p* = 0.05 level.

**Figure 12:**
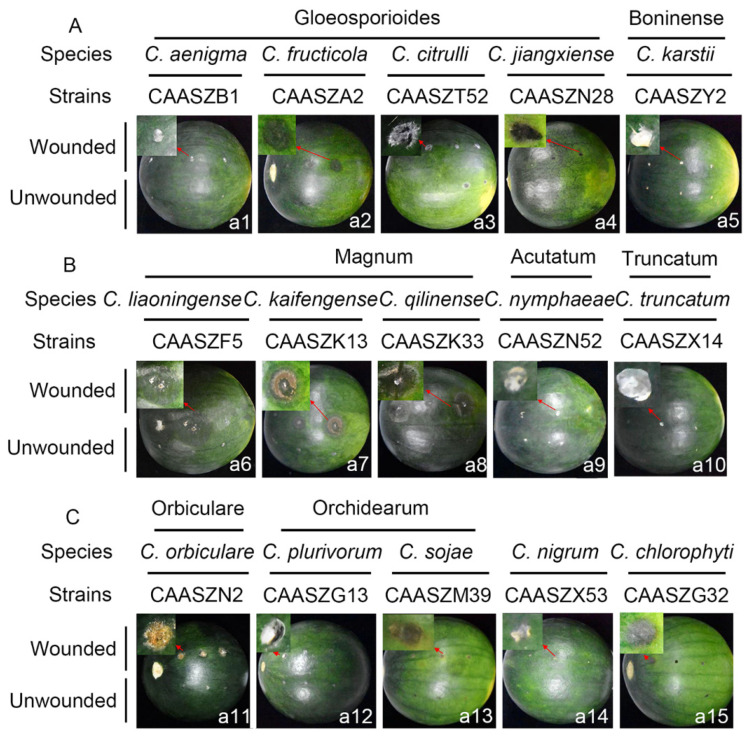
Symptoms of watermelon fruits (*Citrullus. lanatus* cv. Motong) induced by inoculation of spore suspensions of 15 Colletotrichum spp. under unwounded and wounded conditions. The symptoms caused by these species were photographed at 6 dpi. (**a1**–**a15**) Under wounded and unwounded conditions, s symptoms induced by the isolates/species belonging to the *C. gloeosporioides, C. boninense, C. magnum, C. acutatum, C. truncatum, C. orbiculare,* and *C. orchidearum* complexe species or singleton species, respectively. The symptoms induced by the isolates/species belonging to the *C. gloeosporioides* complex and the *C. boninense* complex (**A**), the *C. magnum* complex (**B**), and other complexes or singleton species (**C**), respectively. The inoculation was conducted by dropping 1 × 10^7^ spores (conidia or ascospores) per mL on detached fruits of *Citrullus. lanatus* cv. Motong in three replicates after wounded by pin-pricking each leaf for one time with a sterilized needle (wounded) or kept unwounded (unwounded).

Fifteen representative *Colletotrichum* isolates were selected to prove Koch’s postulates. At 4 dpi, all the *Colletotrichum* isolates developed brown to black lesions symptoms of anthracnose on detached wounded leaves of *Citrullus lanatus* cv. Hongheping inoculated by a spore suspension, and *C. fructicola*, *C. chlorophyte,* and *C. nymphaeae* were unable to infect non-wounded leaves (Figure 10(a2,a10,a15), Table 3). Under wounded conditions, pathogenicity tests demonstrated that *C. kaifengense* (mean ± SD = 32.33 ± 6.35 mm), *C. citrulli* (mean ± SD = 27.89 ± 4.71 mm), *C. qilinense* (mean ± SD = 26.84 ± 9.45 mm), and *C. magnum* (mean ± SD = 24.42 ± 0.17 mm) were highly aggressive on watermelon leaves (Figure 11). However, *C. fructicola*, *C. karstii,* and *C. nymphaeae* showed only weak moderate aggression on watermelon leaves (Figure 12). Under unwounded conditions, *C. qilinense* (isolate CAASZK13) had the highest infection rate at 77.8% (Table 3). No lesions were induced in the control leaves inoculated with sterile water.

Pathogenicity was also accessed on detached watermelon fruits of *Citrullus lanatus* cv. Motong. Under unwounded conditions, *C. aenigma*, *C. fructicola*, *C. nigrum,* and *C. truncatum* did not infect the non-wounded fruits (Table 3). *C. karstii*, *C. orbiculare*, *C. magnum*, and *C. sojae* had the highest infection incidence at 100% (Table 3). Under wounded conditions, there was clear variation in aggression among species. Importantly, *C. qilinense* induced large brown or dark brown lesions and formed concentric rings of conidia at 6 dpi (Figure 12). The isolates of the *C. magnum* species complex, including *C. magnum* (mean ± SD = 40.2 ± 0.3 mm), *C. qilinense* (mean ± SD = 36.7 ± 0.6 mm), and *C. kaifengense* (mean ± SD = 27.0 ± 5.4 mm) induced significantly longer lesions than others (Figure 12).

### 3.5. Taxonomy

Based on DNA sequence data and the morphological characteristics, the 146 isolates were assigned to 15 species, including 8 species reported from watermelon for the first time, and 3 species proved to represent new taxa. The three new species in culture are characterized below.

**Figure 13:**
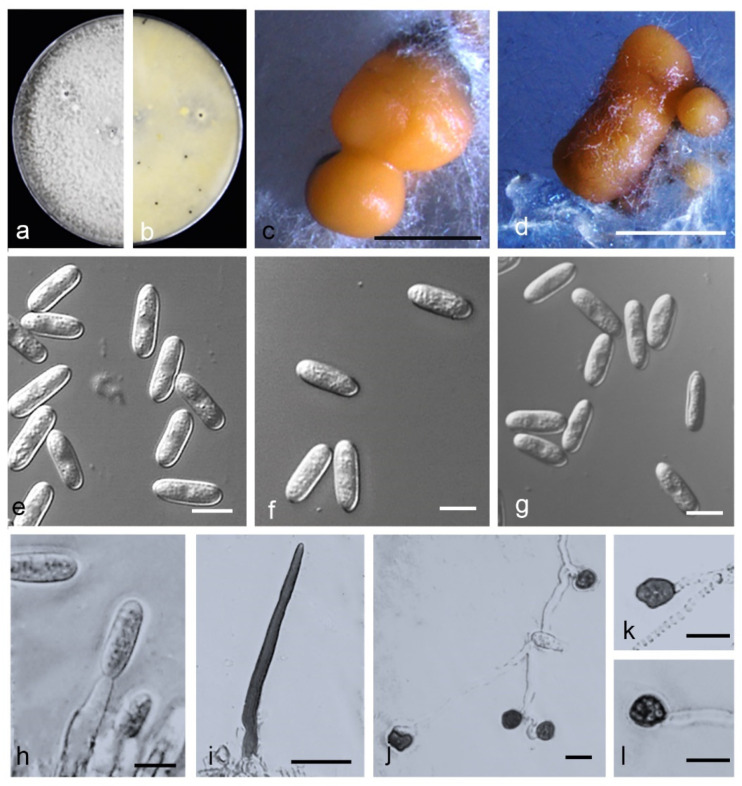
*Colletotrichum citrulli*. (**a**,**b**) Front and back view, respectively, of 9-d-old PDA culture; (**c**,**d**) conidiomata; (**e**–**g**) conidia; (**h**) conidiophores; (**i**) seta; (**j**–**l**) appressoria; (**a**–**q**) isolate CAASZT52; (**a**,**b**) produced on PDA agar medium; (**c**,**e**–**h**) produced on OA agar medium; (**d**,**i**–**l**) produced on SNA agar medium. Scale bars: (**c**,**d**) = 1000 μm; (**i**) = 20 μm; (**h**,**j**–**l**) = 10 μm.

*Colletotrichum citrulli*—Z. Guo & Q.S. Gu, sp. nov. (Figure 13).

MycoBank Number: MB842245.

Etymology: The species epithet is derived from the host plant, *Citrullus lanatus*.

Holotype: China, Henan Province, Zhoukou City, Taikang County, on fruits of *Citrullus lanatus*, June 2020, Z. Guo. Holotype HMAS 351572, Ex-type culture CGMCC 3.20769 = CAASZT52.

*Sexual* morph not observed. *Asexual* morph developed on OA. *Chlamydospores* not observed. *Conidiomata acervular* formed on a cushion of angular cells. *Conidiophores* hyaline to pale brown, aseptate, unbranched. *Conidiogenous cells* hyaline, cylindrical, 10.5–17 × 3–6 µm, opening 1–2 µm. *Conidia* hyaline, smooth-walled, aseptate, apex subacute or obtuse, contents with 1–2 guttules, 15–18.5 × 5–7 µm, mean ± SD = 16.2 ± 0.9 × 5.6 ± 0.5 μm, L/W ratio = 2.9; *Appressoria* single or in groups, medium to dark brown, variable in shape, often navicular to bullet-shaped, circular, clavate, smooth-walled to undulate, 8–12 × 6–10 µm, mean ± SD = 9.9 ± 0.2 × 7.3 ± 0.8 μm, L/W ratio = 1.4.

*Asexual* morph developed on SNA. *Setae* medium to dark brown, smooth to finely verruculose close to the tip, the tip rounded, 1–3- aseptate, 42–79 μm long, base inflated, usually paler, 3–6.5 μm diam. *Conidia* hyaline, smooth-walled, aseptate, apex subacute or obtuse, contents with 1–2 guttules, 14–17.5 × 4–6 µm, mean ± SD = 15.9 ± 1.0 × 5.3 ± 0.4 μm, L/W ratio = 3.0.

Culture characteristics: Colonies on PDA flat with entire margin, aerial mycelium dense, cottony, surface pale cinnamon, covered with irregular grey to black conidiomata, reverse same colors. Colony diam 69.0–70.0 mm in 5 d. Colonies on OA flat with entire margin, aerial mycelium lacking, partly covered with numerous orange conidia, surface white, reverse pale grey, 73.0–73.5 mm in 5 d. Colonies on SNA flat with entire margin, aerial mycelium sparse, surface pale grey, reverse same colors, 66.5–68.0 mm in 5 d.

Notes: isolates of *C. citrulli* are phylogenetically most closely related to *C. nanhuaensis* (Figure 2). They were distinguished by ITS (with 99.15% sequence identity) and *gadph* (97.63%). Furthermore, the PHI test (Φw = 1) did not detect recombination events between *C. citrulli* and *C. nanhuaensis* (Figure 8a). In morphology, *C. citrulli* differs from *C. nanhuaensis* by having longer conidia (14.65–18.4 5 × 4.75–6.6 5 µm vs. 10.5–16 × 4.5–6 µm) [33].

**Figure 14:**
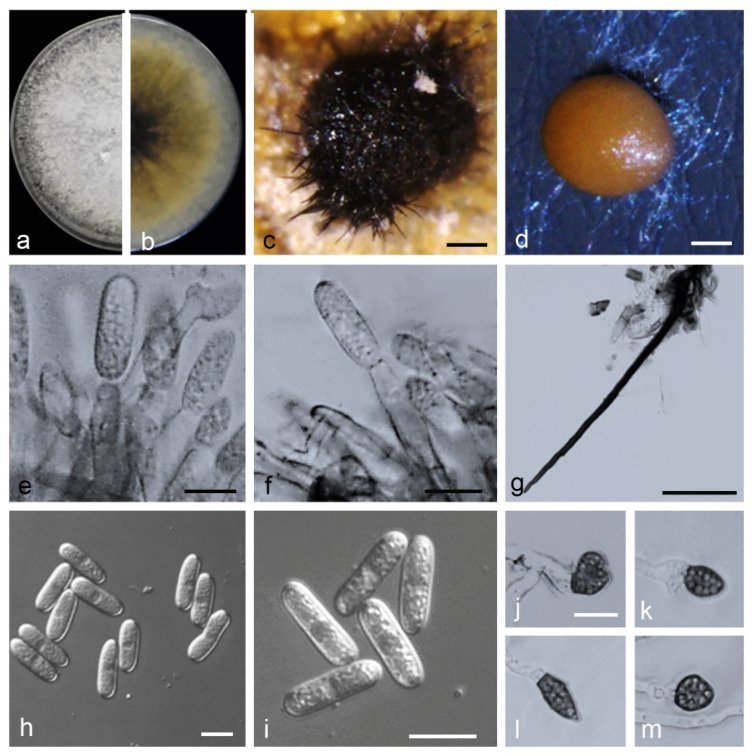
*Colletotrichum kaifengense*. (**a**,**b**) Front and back view, respectively, of 9-d-old PDA culture; (**c**,**d**) conidiomata; (**e**,**f**) conidiophores; (**g**) seta; (**h**,**i**) conidia; (**j**–**m**) appressoria; (**a**–**m**) isolate CAASZK33; (**a**–**c**,**g**) produced on PDA agar medium; (**d**–**f**,**h**–**m**) produced on SNA agar medium). Scale bars: (**c**,**d**) = 100 μm; (**g**) = 50 μm; (**e**,**f**,**h**–**m**) = 10 μm.

*Colletotrichum kaifengense* Z. Guo & Q. S. Gu, sp. nov. (Figure 14).

MycoBank Number: MB842244.

Etymology: Named after Kaifeng, the city in Henan Province, China, where the species was collected.

Holotype: China, Henan Province, Kaifeng City, Jinming County, on fruits of *Citrullus lanatus*, August 2019, Z. Guo. Holotype HMAS 351574, Ex-type culture CGMCC 3.20768 = CAASZK33.

*Sexual* morph not observed. *Asexual* morph developed on PDA. Chlamydospores not observed. *Conidiomata acervular*, conidiophores, and setae formed on a cushion of angular cells. *Conidiophores* hyaline, pale-to medium brown, smooth-walled, aseptate, unbranched. *Conidiogenous cells* hyaline, cylindrical, 11–21 × 4–5 µm. *Setae* medium to dark brown, smooth-walled, the tip acuted, aseptate, 70–153 μm. *Conidia* hyaline, smooth-walled, aseptate, cylindrical, both ends bluntly rounded, 13.5–20 × 4–6.5 µm, mean ± SD = 17.4 ± 1.7× 4.9 ± 0.5 μm, L/W ratio = 3.5. *Appressoria* single or in loose groups, pale to medium brown, bullet-shaped, circular, smooth-walled, spathulate or irregular, 8–14.5 × 5.5–11 µm, mean ± SD = 11.1 ± 1.9× 7.9 ± 1.4μm, L/W ratio = 1.5.

*Asexual* morph developed on OA. *Setae* medium to dark brown, smooth-walled, aseptate, 130.0–200 μm, the tip acute. *Conidia* hyaline, smooth-walled, aseptate, cylindrical, both ends bluntly rounded, 16–20.5 × 4–6.5 µm, mean ± SD = 18.2 ± 1.1× 5.3 ± 0.5 μm, L/W ratio = 3.5.

*Asexual* morph developed on SNA. *Conidiophores,* and Setae formed on hyphae. *Setae* medium to dark brown, smooth-walled, aseptate, 82.5–177.5 μm, the tip acute. *Conidia* hyaline, smooth-walled, aseptate, cylindrical, both ends bluntly rounded, 16–22.5 × 4–5.5 µm, mean ± SD = 20.0 ± 1.2× 4.8 ± 0.4 μm, L/W ratio = 4.2.

Culture characteristics: Colonies on PDA flat with entire margin, aerial mycelium dense, floccose, surface pale grey to white, reverse grey in the center with cinnamon margin, 75.0–76.0 mm in 7 d. Colonies on OA flat with entire margin, aerial mycelium lacking, surface white, covered with small black conidiomata with orange conidial masses, reverse white, covered with irregular black conidiomata, 50.0–52.0 mm in 7 d. Colonies on SNA flat with entire margin, short felty whitish aerial mycelium, surface white, reverse same colors, 50.5–51.5 mm in 7 d.

Notes: isolates of *C. kaifengense* are phylogenetically closely related to *C. magnum* (Figure 6). They were distinguished by *gadph* (93.24%) and *his3* (98.55%). Furthermore, the PHI test (Φw = 1) did not detect recombination events between *C. kaifengense* and *C. magnum* (Figure 8c). They were distinguished by *gadph* (88.14%) and *chs-1* (98.65%). Furthermore, the PHI test (Φw = 1) did not detect recombination events between *C. qilinense* and *C. kaifengense* (Figure 8d).

**Figure 15:**
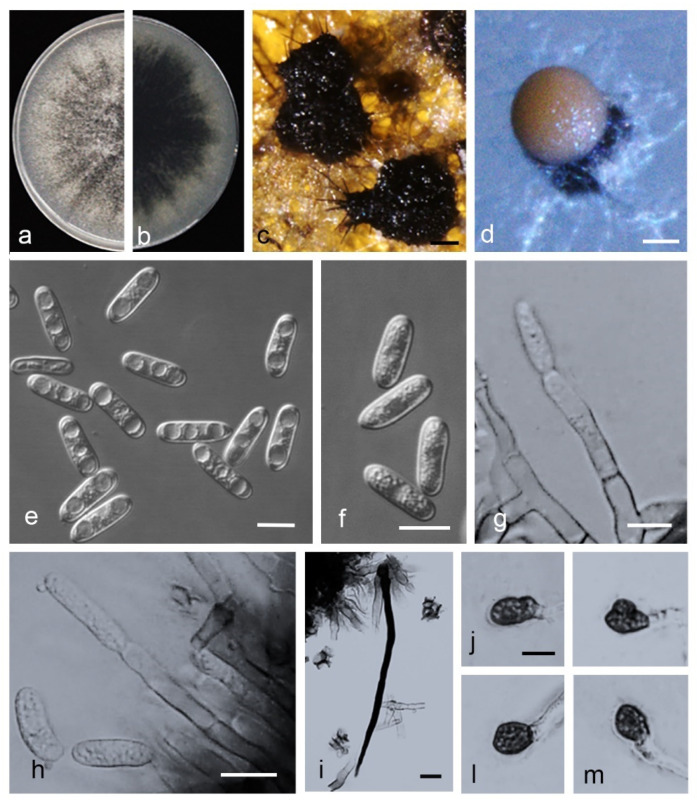
*Colletotrichum qilinense*. (**a**,**b**) Front and back view, respectively, of 9-d-old PDA culture; (**c**,**d**) conidiomata; (**e**,**f**) conidia; (**g**,**h**) conidiophores; (**i**) seta; (**j**–**m**) appressoria; (**a**–**e**,**h**–**m**) isolate CAASZK13; (**f**,**g**) isolate CAASZZ7; (**a**–**c**) produced on PDA agar medium; (**d**–**m**) produced on OA agar medium). Scale bars: (**c**,**d**) = 100 μm; (**i**) = 20 μm; (**e**–**h**,**i**–**m**) = 10 μm; j applies to (**j**–**m**).

*Colletotrichum qilinense* Z. Guo & Q.S. Gu, sp. nov. (Figure 15).

MycoBank Number: MB842247;

Etymology. Referring to the host variety (*Citrullus lanatus* cv. qilinwang) from which the fungus was collected.

Holotype: China, Henan Province, Kaifeng City, Weishi County, on fruits of *Citrullus lanatus*, 20 August 2019, Z. Guo. Holotype HMAS 351573, Ex-type culture CGMCC 3.20767 = CAASZK13).

*Sexual* morph not observed. *Asexual* morph developed on PDA. *Chlamydospores* not observed. *Conidiomata acervular*, conidiophores, and setae formed on a cushion of angular cells. *Conidiophores* hyaline, medium to dark brown, smooth-walled, aseptate, unbranched. *Conidiogenous cells* hyaline, cylindrical, 9–23 × 4–6 µm. *Setae* medium to dark brown, smooth-walled, the tip acuted, 75.5–188 μm. *Conidia* hyaline, smooth-walled, cylindrical, the apex rounded, the base rounded to truncate, 17.5–23 × 5–6 µm, mean ± SD = 19.7 ± 1.4 × 5.4 ± 0.4 μm, L/W ratio = 3.7. *Appressoria* single or in loose groups, medium to dark brown, bullet-shaped, smooth-walled, aseptate, mostly ovoid or ellipsoidal to irregular in outline, 8–16 × 5–11 µm, mean ± SD = 10.6 ± 2.1 × 7.7 ± 1.5 μm, L/W ratio = 1.4.

*Asexual* morph developed on OA. *Setae* medium to dark brown, smooth-walled, the tip acuted, base inflated or not, 96.7–178.4 μm. *Conidia* hyaline, smooth-walled, aseptate or aseptate, cylindrical, the apex rounded, the base rounded to truncate, 14–20 × 5.5–6.5 µm, mean ± SD = 17.6 ± 1.5 × 5.3 ± 0.5 μm, L/W ratio = 3.3.

*Asexual* morph developed on SNA. Conidiophores are directly formed on hyphae. *Setae* medium to dark brown, smooth-walled, the tip acuted, aseptate, 83–203 μm. *Conidia* hyaline, smooth-walled, aseptate, cylindrical, both ends bluntly rounded, 16–20 × 4.5–6 µm, mean ± SD = 17.8 ± 1.0 × 5.1 ± 0.4μm, L/W ratio = 3.5.

Culture characteristics: colonies (CAASZK13) on PDA flat with entire margin, aerial mycelium lacking, floccose, surface dark grey to black, reverse black in center with white margin, 75.50–76.0 mm in 7 d. Colonies on OA flat with entire margin, surface white, partly covered with short felty whitish aerial mycelium, reverse white, covered with irregular black conidiomata, 53.0–54.0 mm in 7 d. Colonies on SNA flat with entire margin, short felty whitish aerial mycelium, surface white, reverse same colors, 51.0–52.0 mm in 7 d.

Notes: isolates of *C. qilinense* are phylogenetically closely related to *C. magnum* (Figure 6). They were distinguished by *gadph* (87.08%), *chs-1* (99.71%), and *act* (96.57%). Furthermore, the PHI test (Φw = 1) did not detect recombination events between *C. qilinense* and *C. magnum* (Figure 8d).

## 4. Discussion

The main objective of this study was to identify the *Colletotrichum* species associated with watermelon in China. We obtained 526 single spore strains from watermelon stems, leaves, and fruits displaying anthracnose symptoms. Based on multi-locus data combined with morphological characteristics we revealed 15 species allocated in 7 species complexes, including gloeosporioides (*C. aenigma*, *C. fructicola*, *C. jiangxiense*, and *C. citrulli*), boninense (*C. karstii*), orbiculare (*C. orbiculare*), orchidearum (*C. plurivorum* and *C. sojae*), magnum (*C. liaoningense*, *C. magnum*, *C. qilinnense,* and *C. kaifengense*), Truncatum (*C. truncatum*), and acutatum (*C. nymphaeae*) and two singleton species (*C. nigrum*, and *C. chlorophyti*). It is the first report of *C. aenigma*, *C. fructicola*, *C. jiangxiense*, *C. plurivorum*, *C. sojae*, *C. magnum*, *C. nymphaeae*, *C. nigrum*, and *C. chlorophyti* causing anthracnose of watermelon. Importantly, this study differentiated three new species, namely *C. citrulli*, *C. qilinnense,* and *C. kaifengense*.

The species complexes of the *Colletotrichum* include several taxa [30,31,32,36,37,38,39,43]. Thus, the complex species related to the 15 *Colletotrichum* species recovered in this study were selected and included in the analyses. In previous studies, although morphological characters delimited some *Colletotrichum* species, some species were difficult to describe due to their morphological variability [25,77]. Thus, in recent studies, the multi-locus phylogenetic analyses combined with morphological characteristics including measurements of setae, conidia, and appressoria, colony characters, and growth rates were able to resolve the complexes and species within *Colletotrichum*. spp [12,16,17,18,78,79]. In this study, most of the *Colletotrichum* species were determined by multi-locus DNA sequence analyses, and some species also exhibited specific morphological characteristics. For example, the growth rate of *C. orbiculare* was significantly lower than that of the other 14 *Colletotrichum* species (Supplementary Figure S1). It is worth noting that the *Colletotrichum* species associated with watermelon differ in their ability to form sexual morphs and setae. Importantly, only four species, *C. aenigma*, *C. fructicola*, *C. karstii*, and *C. plurivorum*, produced ascospores on OA plates (Supplementary Table S3). Additionally, setae of *C. jiangxiense*, *C. nigrum,* and *C. nymphaeae* were not observed on PDA, OA, and SNA plates (Supplementary Table S3).

The prevalence of a *Colletotrichum* species associated with watermelon anthracnose is closely related to the sampling area and plant tissue. For example, some of *Colletotrichum* species, including *C. jiangxiense*, *C. citrulli*, *C. sojae*, *C. magnum*, *C. qilinnense, C. kaifengense*, *C. nymphaeae*, *C. nigrum*, and *C. chlorophyti* associated with watermelon showed restricted distribution. Notably, *C. orbiculare* was isolated from five provinces (Liaoning, Jilin, Henan, Hubei, and Jiangsu), and was dominant in our investigation of the major watermelon-growing regions in China (Figure 9a). It is worth noting that geographical preference was found for *C. aenigma,* in agreement with recent results in China [18].

Some *Colletotrichum* species are host-organ-specific and, for example, predominantly infect leaves of apple and pear [80]. This study revealed that more *Colletotrichum* species infect watermelon leaves than stems or fruits. Three *Colletotrichum* species including *C. aenigma*, *C. sojae*, and *C. nymphaeae* were only isolated from the leaves; *C. chlorophyti* was only isolated from the stems; and five *Colletotrichum* species, including *C. citrulli*, *C. magnum*, *C. qilinnense,* and *C. kaifengense*, were only isolated from fruits (Figure 9c).

Pathogenicity of 15 *Colletotrichum* species isolated from watermelon samples in China was tested on watermelon fruits and leaves. It is worth noting that all *Colletotrichum* species could infect wounded fruits and leaves. However, five *Colletotrichum* species, including *C. aenigma*, *C. fructicola*, *C. magnum*, *C. nigrum,* and *C. truncatum*, did not cause disease symptoms on the non-wounded watermelon fruits. Three *Colletotrichum* species (*C. fructicola*, *C. nymphaeae,* and *C. chlorophyti*) did not cause lesions on the non-wounded leaves (Table 3). This phenomenon is related to inoculation methods and conditions, quiescent infection, and structural defense of the host tissue. In this study, pathogenicity experiments were conducted on wounded or unwounded tissues under extreme conditions of artificial inoculation. It remains to be seen how the symptoms and lesion development are displayed under natural conditions. The quiescent infection is an important feature of *Colletotrichum* spp., and it mainly occurs prior to harvest and eventually leads to fruit rot [81,82,83]. A previous study indicated that the cuticle and epidermis were important as resistance barriers to the initial infection. Wounding can break the quiescent infection and enhance the infectiousness [84].

Different *Colletotrichum* species have varying aggressiveness on the host [17,18,85]. In this study, most isolates belonging to the *C. magnum* species complex showed higher aggressiveness than those of the *C. gloeosporioides*, *C. boninense*, *C. orbiculare, C. orchidearum, C. truncatum*, and *C. acutatum* species complexes (Figure 11). Studies suggest that *C. magnum* caused watermelon anthracnose in USA and chili anthracnose in China [16,32,43]. However, it was never reported on watermelon in China. Thus, this study here represents the first report of *C. magnum* on watermelon in China. *Colletotrichum qilinense* and *C. kaifengense* were found to cause anthracnose on watermelon fruits and were described as new species in this study. Noteworthy, among the 15 representative *Colletotrichum* species in inoculated wounded watermelon leaves, *C. kaifengense* was the most aggressive one (Figure 11).

Previous studies revealed that 15 *Colletotrichum* species were identified from chili fruits with anthracnose symptoms in China, including *C. fioriniae*, *C. fructicola*, *C. gloeosporioides*, *C. scovillei*, *C. truncatum*, *C. conoides*, *C. grossum*, *C. magnum*, *C. aenigma*, *C. cliviae*, *C. endophytica*, *C. hymenocallidis*, *C. incanum*, *C. karstii*, and *C. viniferum* [16,43]. Furthermore, it is interesting that five *Colletotrichum* species, including *C. fructicola*, *C. truncatum*, *C. magnum*, *C. aenigma*, and *C. karstii*, were also isolated from watermelon in this study. *Colletotrichum fructicola* was reported for the first time, associated with anthracnose symptoms from strawberry in USA and Canada [30]. This is the first report of *C. fructicola* to induce anthracnose of watermelon (Figure 2). *Colletotrichum truncatum* was the dominant species on chili in China [16], and it was also found to cause anthracnose on watermelon leaves, stems, and fruits. *Colletotrichum aenigma* was first reported on chili in China, where it was found to cause anthracnose on fruits [16], but this is the first report of *C. aenigma* on *Citrullus lanatus*, where it was found to cause anthracnose on watermelon leaves. *Colletotrichum karstii* was reported causing fruit spots on chili in China [16] and bitter rot on strawberry in the Australia [86]. This is the first report of *C. karstii* in China, which caused watermelon leaf spot.

This study provides useful information about ecology and pathogenicity of 15 *Colletotrichum* spp. involved in watermelon anthracnose. This is of great significance for the control of anthracnose disease in different regions in China.

## 5. Conclusions

This study provides the first summary of 15 *Colletotrichum* spp. associated with watermelon in China. Furthermore, this study presents the first reports of *C. aenigma*, *C. fructicola*, *C. jiangxiense*, *C. plurivorum*, *C. sojae*, *C. nymphaeae*, *C. nigrum*, and *C. chlorophyti*, and three new species (*C. citrulli*, *C. qilinnense,* and *C. kaifengense*), causing anthracnose of watermelon in China.

## Figures and Tables

**Table 1:** A list of *Colletotrichum* isolates collected from *Citrullus lanatus* in China.

Location	Host Tissue	Year	Latitude and Longitude	Number of Isolates
Jiyang, Hainan	leaf and stem	2018	109.58° E, 18.28° N	20
Tongxu, Henan	fruit	2019	114.47° E, 34.48° N	8
Weishi, Henan	fruit	2019	114.29° E, 34.35° N	16
Xiangfu, Henan	fruit	2019	114.35° E, 34.77° N	6
Jinming, Henan	fruit	2019	114.31° E, 34.87° N	19
	fruit	2020		26
Zhongmou, Henan	leaf and stem	2019	113.98° E, 34.71° N	25
	leaf and stem	2020		9
Xinzheng, Henan	fruit	2019	113.74° E, 34.39° N	8
Fengcheng, Liaoning	leaf and stem	2019	124.06° E, 40.45° N	24
Ningbo, Zhejiang	leaf and stem	2019	121.54° E, 29.87° N	18
Taikang, Hennan	fruit	2019	114.62° E, 34.11° N	44
Ganzhou, Jingxi	leaf and stem	2020	115.78° E, 25.60° N	48
Xinxiang, Henan	leaf, stem and fruit	2020	113.85° E, 35.30° N	91
Enshi, Hubei	leaf and stem	2020	109.47° E, 30.29° N	52
Fugou, Henan	fruit	2020	114.39° E, 34.05° N	31
Yancheng, Jiangsu	fruit	2020	120.15° E, 33.34° N	22
Gaoan, Jiangxi	leaf and stem	2020	115.37° E, 28.41° N	35
Tongyu, Jilin	leaf	2020	123.08° E, 44.80° N	24
Total				526

**Table 2:** Nucleotide substitution models used in the phylogenetic analyses.

Gene	ITS	*gadph*	*chs-1*	*his3*	*act*	*tub2*	*gs*
Gloeosporioides clade	GTR + I	HKY + I	K80 + G	GTR + G	GTR + I	SYM + G	–
Boninense clade	SYM + I + G	HKY + G	K80 + G	GTR + I+G	GTR + G	K80 + I	–
Orbiculare clade	GTR	SYM	SYM + I	GTR + G	HKY + G	HKY	GTR + G
Orchidearum clade	GTR + I	HKY	GTR + G	HKY + G	HKY	HKY + I	–
Magnum clade	GTR + G	HKY + G	GTR + I	HKY + I	HKY + G	GTR + G	–
Acutatum clade and other taxa	GTR + I + G	HKY + I + G	GTR + I+G	–	HKY + I + G	HKY + I + G	–

**Table 3:** Incidence of infection on leaves of *Citrullus lanatus* cv. Hongheping and fruit of *Citrullus lanatus* cv. Motong by *Colletotrichum* species.

Species	Strain	Infection Incidence %	
		Leaf Bioassay	Fruit Bioassay
*C.aenigma*	CAASZB1	44.4	0.0
*C.fructicola*	CAASZA2	0.0	0.0
*C. citrullus*	CAASZT52	66.7	66.7
*C.jiangxiense*	CAASZN28	44.4	33.3
*C.karstii*	CAASZY2	44.4	100.0
*C.orbiculare*	CAASZN2	44.4	100.0
*C.plurivorum*	CAASZG13	55.6	66.7
*C.sojae*	CAASZM39	33.3	100.0
*C.magnum*	CAASZF5	66.7	100.0
*C.qilinense*	CAASZK13	77.8	66.7
*C.kaifengense*	CAASZK33	66.7	66.7
*C.nymphaeae*	CAASZN52	0.0	66.7
*C.nigrum*	CAASZX53	44.4	0.0
*C.chlorophyti*	CAASZG32	0.0	33.3
*C.truncatum*	CAASZX14	33.3	0.0

## Data Availability

Alignments generated during the current study are available from TreeBASE (study 29157). All sequence data are available in the NCBI GenBank, following the accession numbers in the manuscript.

## Supplementary Materials

The following supporting information can be downloaded at: https://www.mdpi.com/article/10.3390/jof8080790/s1, Table S1. List of 148 representative isolates of 15 *Colletotrichum* spp. collected from watermelon in China, with details host and location, and Genbank No; Table S2. Strains of the *Colletotrichum* species in this paper with details about host and location, and Genbank No; Table S3. The sizes of seta, conidia, appresoria, and ascospores of the representative isolates of Colletotrichum spp. obtained in this study; Figure S1. The growth rate of the representative isolates of *Colletotrichum* spp. obtained in this study. Data were analyzed with SPSS Statistics 19.0 (WinWrap Basic; http://www.winwrap.com (accessed on 18 December 2021)) by one-way analysis of variance, and means were compared using Duncan’s test at a significance level of *p* = 0.05. Letters over the error bars indicate the significant difference at the *p* = 0.05 level.
