# Supplementary material for: Colletotrichum Species Associated with Anthracnose Disease of Watermelon (Citrullus lanatus) in China
Source: J Fungi (Basel). 2022 Jul 28;8(8):790. doi: 10.3390/jof8080790 (PMC9410023; doi:10.3390/jof8080790)
Supplement: Supplementary file 1 [file jof-08-00790-s001.zip › figure s1.pdf]

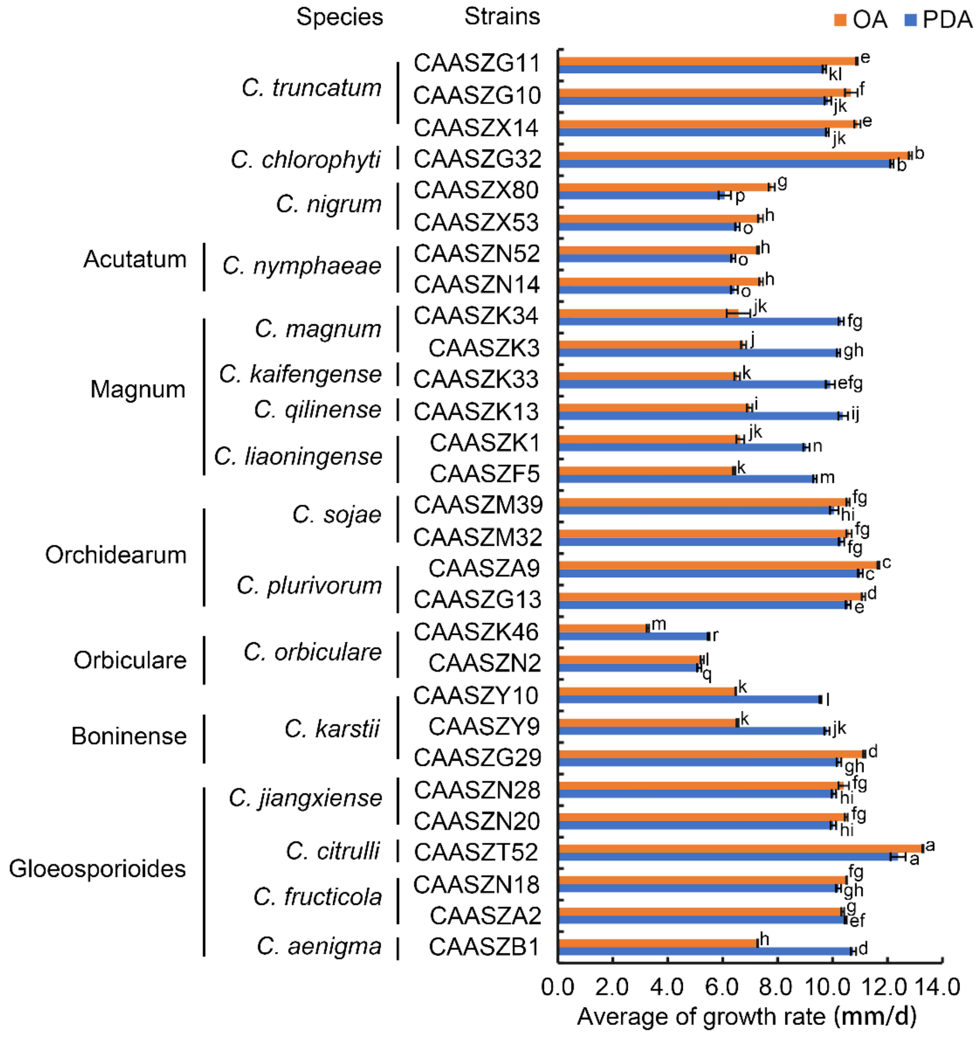

Figure S1. The growth rate of the representative isolates of Colletotrichum spp. obtained in this study. Data were analysed with SPSS Statistics 19.0 (WinWrap Basic; http://www.winwrap.com) by one-way analysis of variance, and means were compared using Duncan's test at a significance level of P = 0.05. Letters over the error bars indicate the significant difference at the P = 0.05 level.
