# Supplementary material for: Colletotrichum Species Associated with Anthracnose Disease of Watermelon (Citrullus lanatus) in China
Source: J Fungi (Basel). 2022 Jul 28;8(8):790. doi: 10.3390/jof8080790 (PMC9410023; doi:10.3390/jof8080790)
Supplement: Supplementary file 1 [file jof-08-00790-s001.zip › Table s1.pdf]

Table S1. List of 148 representative isolates of 15 *Colletotrichum* spp. collected from watermelon in China, with details host and location, and Genbank No.

Species	Isolate No	Host	Location	GenBank No						
				ITS	*gadph*	*chs-1*	*his3*	*act*	*tub2*	*gs*
C. aenigma	CAASZB1	*Citrullus lanatus*, leaf	Ningbo, Zhejiang	MZ475286	OL898946	OL986036	ON479073	OL986216	OL988221	—
	CAASZB7	*Citrullus lanatus*, leaf	Ningbo, Zhejiang	MZ475287	OL898947	OL986037	ON479074	OL986217	OL988222	—
	CAASZB13	*Citrullus lanatus*, leaf	Ningbo, Zhejiang	MZ475288	OL898948	OL986038	ON479075	OL986218	OL988223	—
C. chlorophyti	CAASZG32	*Citrullus lanatus*, stem	Ganzhou, Jiangxi	MZ475145	OL899024	OL986114	—	OL986294	OL988299	—
	CAASZG33	*Citrullus lanatus*, stem	Ganzhou, Jiangxi	MZ475146	OL899025	OL986115	—	OL986295	OL988300	—
C. citrulli	**CAASZT52***	***Citrullus lanatus*, fruit**	**Taikang, Hennan**	**MZ475133**	**OL456685**	**OL901153**	**ON479082**	**OL449283**	**OL456644**	—
	CAASZT54	*Citrullus lanatus*, fruit	Taikang, Hennan	MZ475134	OL456686	OL901154	ON479083	OL449284	OL456645	—
C. fructicola	CAASZA2	*Citrullus lanatus*, leaf	Gaoan, Jiangxi	MZ475198	OL898940	OL986030	ON479077	OL986210	OL988215	—
	CAASZN11	*Citrullus lanatus*, stem	Enshi, Hubei	MZ475153	OL898941	OL986031	ON479080	OL986211	OL988216	—
	CAASZN18	*Citrullus lanatus*, stem	Enshi, Hubei	MZ475155	OL898942	OL986032	ON479079	OL986212	OL988217	—
	CAASZN21	*Citrullus lanatus*, stem	Enshi, Hubei	MZ475157	OL898943	OL986033	ON479078	OL986213	OL988218	—
	CAASZN33	*Citrullus lanatus*, leaf	Enshi, Hubei	MZ475159	OL898944	OL986034	ON479076	OL986214	OL988219	—
	CAASZN5	*Citrullus lanatus*, stem	Enshi, Hubei	MZ475152	OL898945	OL986035	ON479081	OL986215	OL988220	—
C. jiangxiense	CAASZN4	*Citrullus lanatus*, stem	Enshi, Hubei	MZ475151	OL898949	OL986039	ON479084	OL986219	OL988224	—
	CAASZN28	*Citrullus lanatus*, leaf	Enshi, Hubei	MZ475158	OL898950	OL986040	ON479085	OL986220	OL988225	—
C. kaifengense	CAASZK10	*Citrullus lanatus*, fruit	Weishi, Henan	MZ475214	OL456709	OL901177	OM057718	OL449307	OL456668	—
	CAASZK27	*Citrullus lanatus*, fruit	Jinming, Henan	MZ475239	OL456710	OL901178	OM057719	OL449308	OL456669	—
	CAASZK28	*Citrullus lanatus*, fruit	Jinming, Henan	MZ475240	OL456711	OL901179	OM057720	OL449309	OL456670	—
	CAASZK29	*Citrullus lanatus*, fruit	Jinming, Henan	MZ475241	OL456712	OL901180	OM057721	OL449310	OL456671	—
	CAASZK32	*Citrullus lanatus*, fruit	Jinming, Henan	MZ475244	OL456714	OL901182	OM057723	OL449312	OL456673	—
	**CAASZK33***	***Citrullus lanatus*, fruit**	**Jinming, Henan**	**MZ475245**	**OL456715**	**OL901183**	**OM057724**	**OL449313**	**OL456674**	—
C. karstii	CAASZG29	*Citrullus lanatus*, stem	Ganzhou, Jiangxi	MZ475144	OL898951	OL986041	OL986137	OL986221	OL988226	—
	CAASZG43	*Citrullus lanatus*, leaf	Ganzhou, Jiangxi	MZ475148	OL898952	OL986042	OL986138	OL986222	OL988227	—
	CAASZG46	*Citrullus lanatus*, leaf	Ganzhou, Jiangxi	MZ475149	OL898953	OL986043	OL986139	OL986223	OL988228	—
	CAASZY1	*Citrullus lanatus*, leaf	Jiyang, Hainan	MZ475254	OL898954	OL986044	OL986140	OL986224	OL988229	—
	CAASZY10	*Citrullus lanatus*, leaf	Jiyang, Hainan	MZ475263	OL898955	OL986045	OL986141	OL986225	OL988230	—

Table S1. (cont.)

Species	Isolate No	Host	Location	GenBank No						
				ITS	*gadph*	*his*	*his3*	*act*	*tub2*	*gs*
	CAASZY11	*Citrullus lanatus*, leaf	Jiyang, Hainan	MZ475264	OL898956	OL986046	OL986142	OL986226	OL988231	—
	CAASZY12	*Citrullus lanatus*, leaf	Jiyang, Hainan	MZ475265	OL898957	OL986047	OL986143	OL986227	OL988232	—
	CAASZY13	*Citrullus lanatus*, leaf	Jiyang, Hainan	MZ475266	OL898958	OL986048	OL986144	OL986228	OL988233	—
	CAASZY14	*Citrullus lanatus*, leaf	Jiyang, Hainan	MZ475267	OL898959	OL986049	OL986145	OL986229	OL988234	—
	CAASZY15	*Citrullus lanatus*, leaf	Jiyang, Hainan	MZ475268	OL898960	OL986050	OL986146	OL986230	OL988235	—
	CAASZY16	*Citrullus lanatus*, leaf	Jiyang, Hainan	MZ475269	OL898961	OL986051	OL986147	OL986231	OL988236	—
	CAASZY17	*Citrullus lanatus*, leaf	Jiyang, Hainan	MZ475270	OL898962	OL986052	OL986148	OL986232	OL988237	—
	CAASZY18	*Citrullus lanatus*, leaf	Jiyang, Hainan	MZ475271	OL898963	OL986053	OL986149	OL986233	OL988238	—
	CAASZY19	*Citrullus lanatus*, leaf	Jiyang, Hainan	MZ475272	OL898964	OL986054	OL986150	OL986234	OL988239	—
	CAASZY2	*Citrullus lanatus*, leaf	Jiyang, Hainan	MZ475255	OL898965	OL986055	OL986151	OL986235	OL988240	—
	CAASZY20	*Citrullus lanatus*, leaf	Jiyang, Hainan	MZ475273	OL898966	OL986056	OL986152	OL986236	OL988241	—
	CAASZY3	*Citrullus lanatus*, leaf	Jiyang, Hainan	MZ475256	OL898967	OL986057	OL986153	OL986237	OL988242	—
	CAASZY4	*Citrullus lanatus*, leaf	Jiyang, Hainan	MZ475257	OL898968	OL986058	OL986154	OL986238	OL988243	—
	CAASZY5	*Citrullus lanatus*, leaf	Jiyang, Hainan	MZ475258	OL898969	OL986059	OL986155	OL986239	OL988244	—
	CAASZY6	*Citrullus lanatus*, leaf	Jiyang, Hainan	MZ475259	OL898970	OL986060	OL986156	OL986240	OL988245	—
	CAASZY7	*Citrullus lanatus*, leaf	Jiyang, Hainan	MZ475260	OL898971	OL986061	OL986157	OL986241	OL988246	—
	CAASZY8	*Citrullus lanatus*, leaf	Jiyang, Hainan	MZ475261	OL898972	OL986062	OL986158	OL986242	OL988247	—
	CAASZY9	*Citrullus lanatus*, leaf	Jiyang, Hainan	MZ475262	OL898973	OL986063	OL986159	OL986243	OL988248	—
C. magnum	CAASZK4	*Citrullus lanatus*, fruit	Tongxu, Henan	MZ475208	OL899045	OL901141	OM057684	OL986315	OL988320	—
	CAASZF5	*Citrullus lanatus*, fruit	Fugou, Henan	MZ475164	OL456675	OL901143	OM057686	OL449273	OL456634	—
	CAASZF9	*Citrullus lanatus*, fruit	Fugou, Henan	MZ475165	OL456676	OL901144	OM057687	OL449274	OL456635	—
	CAASZF13	*Citrullus lanatus*, fruit	Fugou, Henan	MZ475166	OL456677	OL901145	OM057688	OL449275	OL456636	—
	CAASZK1	*Citrullus lanatus*, fruit	Tongxu, Henan	MZ475205	OL456678	OL901146	OM057689	OL449276	OL456637	—
	CAASZK2	*Citrullus lanatus*, fruit	Tongxu, Henan	MZ475206	OL456679	OL901147	OM057690	OL449277	OL456638	—
	CAASZK5	*Citrullus lanatus*, fruit	Tongxu, Henan	MZ475209	OL456680	OL901148	OM057691	OL449278	OL456639	—
	CAASZK6	*Citrullus lanatus*, fruit	Tongxu, Henan	MZ475210	OL456681	OL901149	OM057692	OL449279	OL456640	—

Table S1. (cont.)

Species	Isolate No	Host	Location	GenBank No						
				ITS	*gadph*	*chs-1*	*his3*	*act*	*tub2*	*gs*
	CAASZK7	*Citrullus lanatus*, fruit	Tongxu, Henan	MZ475211	OL456682	OL901150	OM057693	OL449280	OL456641	—
	CAASZK8	*Citrullus lanatus*, fruit	Tongxu, Henan	MZ475212	OL456683	OL901151	OM057694	OL449281	OL456642	—
	CAASZK9	*Citrullus lanatus*, fruit	Weishi, Henan	MZ475213	OL899046	OL901142	OM057685	OL986316	OL988321	—
	CAASZK3	*Citrullus lanatus*, fruit	Tongxu, Henan	MZ475207	OL899043	OL986133	OL986208	OL986313	OL988318	—
	CAASZK34	*Citrullus lanatus*, fruit	Jinming, Henan	MZ475246	OL899044	OL986134	OL986209	OL986314	OL988319	—
C. nigrum	CAASZX19	*Citrullus lanatus*, stem	Xinxiang, Henan	MZ475182	OL899026	OL986116	—	OL986296	OL988301	—
	CAASZX53	*Citrullus lanatus*, stem	Xinxiang, Henan	MZ475187	OL899027	OL986117	—	OL986297	OL988302	—
	CAASZX80	*Citrullus lanatus*, leaf	Xinxiang, Henan	MZ475191	OL899028	OL986118	—	OL986298	OL988303	—
C. nymphaeae	CAASZN14	*Citrullus lanatus*, leaf	Enshi, Hubei	MZ475154	OL899022	OL986112	—	OL986292	OL988297	—
	CAASZN52	*Citrullus lanatus*, leaf	Enshi, Hubei	MZ475161	OL899023	OL986113	—	OL986293	OL988298	—
C. orbiculare	CAASZC13	*Citrullus lanatus*, leaf	Tongyu, Jilin	MZ475136	OL898974	OL986064	OL986160	OL986244	OL988249	OM057725
	CAASZC19	*Citrullus lanatus*, leaf	Tongyu, Jilin	MZ475137	OL898975	OL986065	OL986161	OL986245	OL988250	OM057726
	CAASZC7	*Citrullus lanatus*, stem	Tongyu, Jilin	MZ475135	OL898976	OL986066	OL986162	OL986246	OL988251	OM057727
	CAASZD1	*Citrullus lanatus*, stem	Fengcheng, Liaoning	MZ475279	OL898977	OL986067	OL986163	OL986247	OL988252	OM057728
	CAASZD13	*Citrullus lanatus*, leaf	Fengcheng, Liaoning	MZ475281	OL898978	OL986068	OL986164	OL986248	OL988253	OM057729
	CAASZD19	*Citrullus lanatus*, leaf	Fengcheng, Liaoning	MZ475282	OL898979	OL986069	OL986165	OL986249	OL988254	OM057730
	CAASZD7	*Citrullus lanatus*, stem	Fengcheng, Liaoning	MZ475280	OL898980	OL986070	OL986166	OL986250	OL988255	OM057731
	CAASZF27	*Citrullus lanatus*, fruit	Fugou, Henan	MZ475169	OL898981	OL986071	OL986167	OL986251	OL988256	OM057732
	CAASZF3	*Citrullus lanatus*, fruit	Fugou, Henan	MZ475162	OL898982	OL986072	OL986168	OL986252	OL988257	—
	CAASZF4	*Citrullus lanatus*, fruit	Fugou, Henan	MZ475163	OL898983	OL986073	OL986169	OL986253	OL988258	OM057733
	CAASZJ1	*Citrullus lanatus*, fruit	Yancheng, Jiangsu	MZ475170	OL898984	OL986074	OL986170	OL986254	OL988259	OM057734
	CAASZJ2	*Citrullus lanatus*, fruit	Yancheng, Jiangsu	MZ475171	OL898985	OL986075	OL986171	OL986255	OL988260	OM057735
	CAASZJ20	*Citrullus lanatus*, fruit	Yancheng, Jiangsu	MZ475173	OL898986	OL986076	OL986172	OL986256	OL988261	OM057736
	CAASZJ8	*Citrullus lanatus*, fruit	Yancheng, Jiangsu	MZ475172	OL898987	OL986077	OL986173	OL986257	OL988262	OM057737
	CAASZK42	*Citrullus lanatus*, fruit	Xiangfu, Henan	MZ475227	OL898988	OL986078	OL986174	OL986258	OL988263	OM057738
	CAASZK43	*Citrullus lanatus*, fruit	Xiangfu, Henan	MZ475228	OL898989	OL986079	OL986175	OL986259	OL988264	OM057739

Table S1. (cont.)

Species	Isolate No	Host	Location	GenBank No						
				ITS	*gadph*	*chs-1*	*his3*	*act*	*tub2*	*gs*
	CAASZK44	*Citrullus lanatus*, fruit	Xiangfu, Henan	MZ475229	OL898990	OL986080	OL986176	OL986260	OL988265	OM057740
	CAASZK45	*Citrullus lanatus*, fruit	Xiangfu, Henan	MZ475230	OL898991	OL986081	OL986177	OL986261	OL988266	OM057741
	CAASZK46	*Citrullus lanatus*, fruit	Xiangfu, Henan	MZ475231	OL898992	OL986082	OL986178	OL986262	OL988267	OM057742
	CAASZK47	*Citrullus lanatus*, fruit	Xiangfu, Henan	MZ475232	OL898993	OL986083	OL986179	OL986263	OL988268	OM057743
	CAASZK48	*Citrullus lanatus*, fruit	Xiangfu, Henan	MZ475233	OL898994	OL986084	OL986180	OL986264	OL988269	OM057744
	CAASZK54	*Citrullus lanatus*, fruit	Xiangfu, Henan	MZ475174	OL898995	OL986085	OL986181	OL986265	OL988270	—
	CAASZK55	*Citrullus lanatus*, fruit	Xiangfu, Henan	MZ475175	OL898996	OL986086	OL986182	OL986266	OL988271	OM057745
	CAASZK67	*Citrullus lanatus*, fruit	Xiangfu, Henan	MZ475176	OL898997	OL986087	OL986183	OL986267	OL988272	—
	CAASZM1	*Citrullus lanatus*, leaf	Zhongmou, Henan	MZ475274	OL898998	OL986088	OL986184	OL986268	OL988273	—
	CAASZM19	*Citrullus lanatus*, leaf	Zhongmou, Henan	MZ475277	OL898999	OL986089	OL986185	OL986269	OL988274	—
	CAASZN2	*Citrullus lanatus*, stem	Enshi, Hubei	MZ475150	OL899000	OL986090	OL986186	OL986270	OL988275	OM057746
	CAASZN20	*Citrullus lanatus*, stem	Enshi, Hubei	MZ475156	OL899001	OL986091	OL986187	OL986271	OL988276	—
	CAASZN46	*Citrullus lanatus*, leaf	Enshi, Hubei	MZ475160	OL899002	OL986092	OL986188	OL986272	OL988277	OM057747
	CAASZT40	*Citrullus lanatus*, fruit	Taikang, Hennan	MZ475129	OL899003	OL986093	OL986189	OL986273	OL988278	OM057748
	CAASZT46	*Citrullus lanatus*, fruit	Taikang, Hennan	MZ475131	OL899004	OL986094	OL986190	OL986274	OL988279	OM057749
	CAASZX1	*Citrullus lanatus*, fruit	Xinxiang, Henan	MZ475177	OL899005	OL986095	OL986191	OL986275	OL988280	—
	CAASZX3	*Citrullus lanatus*, fruit	Xinxiang, Henan	MZ475178	OL899006	OL986096	OL986192	OL986276	OL988281	OM057750
	CAASZX5	*Citrullus lanatus*, fruit	Gaoan, Jiangxi	MZ475179	OL899007	OL986097	OL986193	OL986277	OL988282	OM057751
C. plurivorum	CAASZA10	*Citrullus lanatus*, leaf	Gaoan, Jiangxi	MZ475200	OL899008	OL986098	OL986194	OL986278	OL988283	—
	CAASZA14	*Citrullus lanatus*, leaf	Gaoan, Jiangxi	MZ475201	OL899009	OL986099	OL986195	OL986279	OL988284	—
	CAASZA15	*Citrullus lanatus*, leaf	Gaoan, Jiangxi	MZ475202	OL899010	OL986100	OL986196	OL986280	OL988285	—
	CAASZA20	*Citrullus lanatus*, leaf	Gaoan, Jiangxi	MZ475203	OL899011	OL986101	OL986197	OL986281	OL988286	—
	CAASZA30	*Citrullus lanatus*, leaf	Gaoan, Jiangxi	MZ475204	OL899012	OL986102	OL986198	OL986282	OL988287	—
	CAASZA9	*Citrullus lanatus*, leaf	Gaoan, Jiangxi	MZ475199	OL899013	OL986103	OL986199	OL986283	OL988288	—
	CAASZG13	*Citrullus lanatus*, stem	Ganzhou, Jiangxi	MZ475142	OL899014	OL986104	OL986200	OL986284	OL988289	—
	CAASZG2	*Citrullus lanatus*, stem	Ganzhou, Jiangxi	MZ475138	OL899015	OL986105	OL986201	OL986285	OL988290	—

Table S1. (cont.)

Species	Isolate No	Host	Location	GenBank No						
				ITS	*gadph*	*chs-1*	*his3*	*act*	*tub2*	*gs*
	CAASZG5	*Citrullus lanatus*, stem	Ganzhou, Jiangxi	MZ475139	OL899016	OL986106	OL986202	OL986286	OL988291	—
C. qilinense	CAASZT1	*Citrullus lanatus*, fruit	Taikang, Hennan	MZ475124	OL456687	OL901155	OM057696	OL449285	OL456646	—
	CAASZT3	*Citrullus lanatus*, fruit	Taikang, Hennan	MZ475125	OL456688	OL901156	OM057697	OL449286	OL456647	—
	CAASZT5	*Citrullus lanatus*, fruit	Taikang, Hennan	MZ475126	OL456689	OL901157	OM057698	OL449287	OL456648	—
	CAASZT45	*Citrullus lanatus*, fruit	Taikang, Hennan	MZ475130	OL456690	OL901158	OM057699	OL449288	OL456649	—
	CAASZT47	*Citrullus lanatus*, fruit	Taikang, Hennan	MZ475132	OL456691	OL901159	OM057700	OL449289	OL456650	—
	CAASZK11	*Citrullus lanatus*, fruit	Weishi, Henan	MZ475215	OL456692	OL901160	OM057701	OL449290	OL456651	—
	CAASZK12	*Citrullus lanatus*, fruit	Weishi, Henan	MZ475216	OL456693	OL901161	OM057702	OL449291	OL456652	—
	**CAASZK13***	***Citrullus lanatus*, fruit**	**Weishi, Henan**	**MZ475217**	**OL456694**	**OL901162**	**OM057703**	**OL449292**	**OL456653**	—
	CAASZK14	*Citrullus lanatus*, fruit	Weishi, Henan	MZ475218	OL456695	OL901163	OM057704	OL449293	OL456654	—
	CAASZK15	*Citrullus lanatus*, fruit	Weishi, Henan	MZ475219	OL456696	OL901164	OM057705	OL449294	OL456655	—
	CAASZK16	*Citrullus lanatus*, fruit	Weishi, Henan	MZ475220	OL456697	OL901165	OM057706	OL449295	OL456656	—
	CAASZK23	*Citrullus lanatus*, fruit	Jinming, Henan	MZ475235	OL456698	OL901166	OM057707	OL449296	OL456657	—
	CAASZK24	*Citrullus lanatus*, fruit	Jinming, Henan	MZ475236	OL456699	OL901167	OM057708	OL449297	OL456658	—
	CAASZK25	*Citrullus lanatus*, fruit	Jinming, Henan	MZ475237	OL456700	OL901168	OM057709	OL449298	OL456659	—
	CAASZK26	*Citrullus lanatus*, fruit	Jinming, Henan	MZ475238	OL456701	OL901169	OM057710	OL449299	OL456660	—
	CAASZK30	*Citrullus lanatus*, fruit	Jinming, Henan	MZ475242	OL456702	OL901170	OM057711	OL449300	OL456661	—
	CAASZK37	*Citrullus lanatus*, fruit	Jinming, Henan	MZ475249	OL456703	OL901171	OM057712	OL449301	OL456662	—
	CAASZM7	*Citrullus lanatus*, leaf	Zhongmou, Henan	MZ475275	OL456704	OL901172	OM057713	OL449302	OL456663	—
	CAASZM13	*Citrullus lanatus*, leaf	Zhongmou, Henan	MZ475276	OL456705	OL901173	OM057714	OL449303	OL456664	—
	CAASZZ1	*Citrullus lanatus*, fruit	Xinzheng, Henan	MZ475283	OL456706	OL901174	OM057715	OL449304	OL456665	—
	CAASZZ7	*Citrullus lanatus*, fruit	Xinzheng, Henan	MZ475284	OL456707	OL901175	OM057716	OL449305	OL456666	—
	CAASZZ13	*Citrullus lanatus*, fruit	Xinzheng, Henan	MZ475285	OL456708	OL901176	OM057717	OL449306	OL456667	—
C. sojae	CAASZM31	*Citrullus lanatus, leaf*	Zhongmou, Henan	MZ475193	OL899017	OL986107	OL986203	OL986287	OL988292	—
	CAASZM32	*Citrullus lanatus*, leaf	Zhongmou, Henan	MZ475194	OL899018	OL986108	OL986204	OL986288	OL988293	—
	CAASZM37	*Citrullus lanatus*, leaf	Zhongmou, Henan	MZ475195	OL899019	OL986109	OL986205	OL986289	OL988294	—

Table S1. (cont.)

Species	Isolate No	Host	Location	GenBank No						
				ITS	*gadph*	*chs-1*	*his3*	*act*	*tub2*	*gs*
	CAASZM38	*Citrullus lanatus*, leaf	Zhongmou, Henan	MZ475196	OL899020	OL986110	OL986206	OL986290	OL988295	—
	CAASZM39	*Citrullus lanatus*, leaf	Zhongmou, Henan	MZ475197	OL899021	OL986111	OL986207	OL986291	OL988296	—
C. truncatum	CAASZG7	*Citrullus lanatus*, leaf	Ganzhou, Jiangxi	MZ475140	OL899029	OL986119	—	OL986299	OL988304	—
	CAASZG10	*Citrullus lanatus*, leaf	Ganzhou, Jiangxi	MZ475141	OL899030	OL986120	—	OL986300	OL988305	—
	CAASZG21	*Citrullus lanatus*, stem	Ganzhou, Jiangxi	MZ475143	OL899031	OL986121	—	OL986301	OL988306	—
	CAASZG41	*Citrullus lanatus*, stem	Ganzhou, Jiangxi	MZ475147	OL899032	OL986122	—	OL986302	OL988307	—
	CAASZX13	*Citrullus lanatus*, leaf	Xinxiang, Henan	MZ475180	OL899033	OL986123	—	OL986303	OL988308	—
	CAASZX14	*Citrullus lanatus*, leaf	Xinxiang, Henan	MZ475181	OL899034	OL986124	—	OL986304	OL988309	—
	CAASZX27	*Citrullus lanatus*, leaf	Xinxiang, Henan	MZ475183	OL899035	OL986125	—	OL986305	OL988310	—
	CAASZX47	*Citrullus lanatus*, leaf	Xinxiang, Henan	OL454189	OL899036	OL986126	—	OL986306	OL988311	—
	CAASZX35	*Citrullus lanatus*, leaf	Xinxiang, Henan	MZ475185	OL899037	OL986127	—	OL986307	OL988312	—
	CAASZX45	*Citrullus lanatus*, leaf	Xinxiang, Henan	MZ475186	OL899038	OL986128	—	OL986308	OL988313	—
	CAASZX57	*Citrullus lanatus*, leaf	Xinxiang, Henan	MZ475188	OL899039	OL986129	—	OL986309	OL988314	—
	CAASZX58	*Citrullus lanatus*, leaf	Xinxiang, Henan	MZ475189	OL899040	OL986130	—	OL986310	OL988315	—
	CAASZX65	*Citrullus lanatus*, leaf	Xinxiang, Henan	MZ475190	OL899041	OL986131	—	OL986311	OL988316	—
	CAASZX88	*Citrullus lanatus*, leaf	Xinxiang, Henan	MZ475192	OL899042	OL986132	—	OL986312	OL988317	—

* = ex—type or authentic culture, (*) = ex—type or authentic culture of synonymised taxon,and all are in bold font.

[2] ITS: internal transcribed spacers and intervening 5.8S nrDNA; GAPDH: partial glyceraldehyde-3-phosphate dehydrogenase gene; CHS-1: partial chitin synthase-1 gene; HIS: partial histone H3 gene; ACT: partial actin gene; TUB2: partial beta-tubulin gene; GS: intron 2 glutamine synthetase.
