# Supplementary material for: Colletotrichum Species Associated with Anthracnose Disease of Watermelon (Citrullus lanatus) in China
Source: J Fungi (Basel). 2022 Jul 28;8(8):790. doi: 10.3390/jof8080790 (PMC9410023; doi:10.3390/jof8080790)
Supplement: Supplementary file 1 [file jof-08-00790-s001.zip › Table s2.pdf]

Table S2. Strains of the *Colletotrichum* species in this paper with details about host and location, and Genbank No

Species	Accession No[1]	Host	Location	GenBank No[2]						
				ITS	*gadph*	*chs-1*	*his3*	*act1*	*tub2*	*gs*
C. acutatum	CBS 144.29	*Capsicum annuum*, fruit	Sri Lanka	JQ948401	JQ948732	JQ949062	—	JQ949722	JQ950052	—
	__CBS 112996*__	__*Carica papaya*__	__Australia__	__JQ005776__	__JQ948677__	__JQ005797__	—	__JQ005839__	__JQ005860__	—
C. aenigma	__ICMP 18608*__	__Persea americana__	__Israel__	__JX010244__	__JX010044__	__JX009774__	—	__JX009443__	__JX010389__	—
	ICMP 18686	*Pyrus pyrifolia*	Japan	JX010243	JX009913	JX009789	—	JX009519	JX010390	—
C. aeschynomenes	__ICMP 17673*__	__*Aeschynomene virginica*__	__USA__	__JX010176__	__JX009930__	__JX009799__	—	__JX009483__	__JX010392__	—
C. alatae	__CBS 304.67*__	__*Dioscorea alata*__	__India__	__JX010190__	__JX009990__	__JX009837__	—	__JX009471__	__JX010383__	—
C. alienum	__ICMP 12071*__	__*Malus domestica*__	__New Zealand__	__JX010251__	__JX010028__	__JX009882__	—	__JX009572__	__JX010411__	—
C. annellatum	__CH1*__	__*Hevea brasiliensis*, leaf__	__Colombia__	__JQ005222__	__JQ005309__	__JQ005396__	__JQ005483__	__JQ005570__	__JQ005656__	—
C. aotearoa	__ICMP 18537*__	__*Coprosma* sp.__	__New Zealand__	__JX010205__	__JX010005__	__JX009853__	—	__JX009564__	__JX010420__	—
C. arecicola	__CGMCC 3.19667*__	__*Areca catechu*__	__China__	__MK914635__	__MK935455__	__MK935541__	—	__MK935374__	__MK935498__	—
C. artocarpicola	__MFLUCC 18-1167*__	__*Artocarpus heterophyllus*__	__Thailand__	__MN415991__	__MN435568__	__MN435569__	—	__MN435570__	__MN435567__	—
C. asianum	__ICMP 18580*__	__*Coffea arabica*__	__Thailand__	__FJ972612__	__JX010053__	__JX009867__	—	__JX009584__	__JX010406__	—
C. beeveri	__ICMP 18594*__	__*Brachyglottis repanda*__	__New Zealand__	__JQ005171__	__JQ005258__	__JX009842__	__JQ005432__	__JQ005519__	__JQ005605__	—
C. bidentis	__COAD 1020*__	__Bidens subalternans__	__Brazil__	__KF178481__	__KF178506__	__JX009843__	__KF178554__	__KF178578__	__KF178602__	__KF178627__
C. boninense	CBS 128526	*Dacrycarpus dacrydioides*, leaf endophyte	New Zealand	JQ005162	JQ005249	JX009844	—	JQ005510	JQ005596	—
	__MAFF 305972*__	__*Crinum asiaticum* var. sinicum__	__Japan__	__JQ005153__	__JQ005240__	__JX009845__	__JQ005414__	__JQ005501__	__JQ005588__	—
	CBS 128526	*Dacrycarpus dacrydioides*, leaf	New Zealand	JQ005162	JQ005249	JX009846	JQ005423	JQ005510	JQ005596	—
	CBS 129831	*Leucospermum* sp.	Australia	JQ005161	JQ005248	JX009847	JQ005422	JQ005509	JQ005595	—
C. brasiliense	__PAS12*__	__*Passiflora edulis*, fruit anthracnose__	__Brazil__	__JQ005235__	__JQ005322__	__JX009848__	__JQ005496__	__JQ005583__	__JQ005669__	—
C. brassicicola	__LYN 16331*__	__*Brassica oleracea* var. gemmifera, leaf__	__New Zealand__	__JQ005172__	__JQ005259__	__JX009849__	__JQ005433__	__JQ005520__	__JQ005606__	—
C. brevisporum	__BCC 38876*__	__*Neoregalia* sp.__	__Thailand__	__JN050238__	__JN050227__	__JX009850__	—	__JN050216__	__JN050244__	—
	CBS 129958	*Anthurium* sp.	Thailand	MG600763	MG600823	JX009851	MG600909	MG600967	MG601030	—
C. cacao	__CBS 119297*__	__*Theobroma cacao*__	__Costa Rica__	__MG600772__	__MG600832__	__JX009852__	__MG600916__	__MG600976__	__MG601039__	—
C. camelliae	__CGMCC 3.14925*__	__*Camellia sinensis*__	__China__	__KJ955081__	__KJ954782__	__MZ799255__	__MZ673847__	__KJ954363__	__KJ955230__	—
C. carthami	__SAPA100011*__	__*Carthamus tinctorium*__	__Japan__	__AB696998__	—	__JX009853__	—	—	__AB696992__	—
C. cattleyicola	__CBS 170.49*__	__*Cattleya* sp.__	__Belgium__	__MG600758__	__MG600819__	__JX009854__	__MG600905__	__MG600963__	__MG601025__	—
C. chlorophyti	__IMI 103806*__	__*Chlorophytum* sp.__	__India__	__GU227894__	__GU228286__	__JX009855__	—	__GU227992__	__GU228188__	—
	CBS 142.79	*Stylosanthes hamata*	Australia	GU227895	GU228287	JX009856	—	GU227993	GU228189	—

Table S2. (cont.)

Species	Accession No[1]	Host	Location	GenBank No[2]						
				ITS	*gadph*	*chs-1*	*his3*	*act1*	*tub2*	*gs*
C. changpingense	**CGMCC 3.17582***	***Rhizome of Fragaria* × *ananass***	**China**	**KP683152**	**KP683152**	**KP852449**	—	**KP683093**	**MZ673952**	—
C. chrysanthemi	CBS 126519	*Chrysanthemum coronarium*, vascular discoloration	Netherlands	JQ948272	JQ948602	JQ948933	—	JQ949593	JQ949923	
C. chrysophilum	**CMM 4268***	***Musa sp.***	**Brazil**	**KX094252**	**KX094183**	**KX094083**	—	**KX093982**	**KX094285**	—
C. cigarro	**ICMP 18539***	***Olea europaea***	**Australia**	**JX010230**	**JX009966**	**JX009800**	**MZ673837**	**JX009523**	**JX010434**	—
C. circinans	**CBS 221.81***	***Allium cepa***	**Serbia**	**GU227855**	**GU228247**	**JX009858**	—	**GU227953**	**GU228149**	—
C. clidemiae	**ICMP 18658***	***Clidemia hirta***	**USA, Hawaii**	**JX010265**	**JX009989**	**JX009859**	—	**JX009537**	**JX010438**	—
C. cliviicola	**CBS 125375***	***Clivia miniata***	**China**	**MG600733**	**MG600795**	**JX009860**	**MG600892**	**MG600939**	**MG601000**	—
	CSSS2	*Clivia miniata*	China	GU109480	GU085868	JX009861	—	GU085862	GU085870	—
	CBS 133705	*Clivia* sp.	South Africa	MG600732	MG600794	JX009862	MG600891	MG600938	MG600999	—
C. cobbittiense	**BRIP 66219***	***Cordyline stricta* × *C. australis***	**Australia**	**MH087016**	**MH094133**	**MH094135**	**MH094136**	**MH094134**	**MH094137**	—
C. coccodes	CBS 164.49	*Solanum tuberosum*	Netherlands	JQ005775	HM171672	JX009863	—	JQ005838	JQ005859	—
	**CBS 369.75***	***Solanum tuberosum***	**Netherlands**	**HM171679**	**HM171667**	**JX009864**	—	**JX546681**	**HM171673**	—
C. colombiense	**G2***	***Passiflora edulis*, leaf**	**Colombia**	**JQ005174**	**JQ005261**	**JX009865**	**JQ005435**	**JQ005522**	**JQ005608**	—
C. conoides	**CGMCC 3.17615***	***Capsicum annuum***	**China**	**KP890168**	**KP890162**	**JX009866**	—	**KP890144**	**KP890174**	—
	CAUG33	*Capsicum annuum*	China	KP890169	KP890163	JX009867	—	KP890145	KP890175	—
	CAUG34	*Capsicum annuum*	China	KP890170	KP890164	JX009868	—	KP890146	KP890176	—
C. constrictum	**ICMP 12941***	***Citrus limon*, fruit**	**New Zealand**	**JQ005238**	**JQ005325**	**JX009869**	**JQ005499**	**JQ005586**	**JQ005672**	—
C. cordylinicola	**MFLUCC 090551***	***Cordyline fruticosa***	**Thailand**	**JX010226**	**JX009975**	**JX009870**	—	**HM470235**	**JX010440**	—
C. cosmi	**CBS 853.73***	***Cosmos* sp., seed**	**Netherlands**	**JQ948274**	**JQ948604**	**JX009871**	—	**JQ949595**	**JQ949925**	—
C. cymbidiicola	**IMI 347923***	***Cymbidium* sp., leaf**	**Australia**	**JQ005166**	**JQ005253**	**JX009872**	**JQ005427**	**JQ005514**	**JQ005600**	—
C. dacrycarpi	**ICMP 19107***	***Dacrycarpus dacrydioides*, leaf**	**New Zealand**	**JQ005236**	**JQ005323**	**JX009873**	**JQ005497**	**JQ005584**	**JQ005670**	—
C. dimorphum	YMF1.07303	*Ageratina adenophora*	China	OK030866	OK513669	OK513565	—	OK513605	OK513635	—
	**CGMCC 3.16083**	***Ageratina adenophora***	**China**	**OK030867**	**OK513670**	**OK513566**	—	**OK513606**	**OK513636**	—
C. dracaenophilum	**CBS 118199***	***Dracaena sanderana***	**China**	**JX519222**	**JX546707**	**JX009874**	—	**JX519238**	**JX519247**	—
C. endophytica	**LC0324***	***Pennisetu purpureum***	**Thailand**	**KC633854**	**KC832854**	**JX009875**	**MZ673839**	**KF306258**	**MZ673954**	—
C. fioriniae	CBS 127601	*Mangifera indica*, stem	Australia	JQ948311	JQ948641	JX009876	—	JQ949632	JQ949962	—
	**CBS 128517***	***Fiorinia* sp.**	**USA**	**JQ948292**	**JQ948622**	**JX009877**	—	**JQ949613**	**JQ949943**	—
C. fructi	**CBS 346.37***	***Malus sylvestris***	**USA**	**GU227844**	**GU228236**	**JX009878**	—	**GU227942**	**GU228138**	—
C. fructicola	ICMP 18613	*Limonium sinuatum*	Israel	JX010167	JX009998	JX009879	—	JX009491	JX010388	—
	ICMP 18120	*Dioscorea alata*	Nigeria	JX010182	JX010041	JX009880	—	JX009436	JX009436	—
	CBS 125395	*Theobroma cacao*	Panama	JX010172	JX009992	JX009881	—	JX009543	JX010408	—

Table S2. (cont.)

Species	Accession No[1]	Host	Location	GenBank No[2]						
				ITS	*gadph*	*chs-1*	*his3*	*act1*	*tub2*	*gs*
	**ICMP 18581***	***Coffea arabica***	**Thailand**	**JX010165**	**JX010033**	**JX009882**	**—**	**FJ907426**	**JX010405**	**—**
	ICMP 18727	*Fragaria × ananassa*	USA	JX010179	JX010035	**JX009883**	—	JX009565	JX010394	—
	CBS 125397	*Tetragastris panamensis*	Panama	JX010173	JX010032	JX009884	—	JX009581	JX010409	—
	**CBS 238.49(*)**	**Ficus edulis**	**Germany**	**JX010181**	**X009923**	**JX009885**	**—**	**JX009495**	**JX010400**	**—**
C. fructivorum	**CBS 133125***	***Vaccinium macrocarpon***	**USA**	**JX145145**	**MZ664047**	**JX009886**	**—**	**MZ664126**	**JX145196**	**—**
C. gloeosporioides	**IMI 356878***	***Citrus sinensis***	**Italy**	**JX010152**	**JX010056**	**JX009887**	**—**	**JX009531**	**JX010445**	**—**
	ICMP 12939	*Citrus* sp.	New Zealand	JX010149	JX009931	JX009888	—	JX009462	—	—
	ICMP 12938	*Citrus sinensis*	New Zealand	JX010147	JX009935	JX009889	—	JX009560	—	—
	ICMP 18694	*Mangifera indica*	South Africa	JX010155	JX009980	JX009890	—	JX009481	—	—
	ICMP 18695	*Citrus* sp.	USA	JX010153	JX009979	JX009891	—	JX009494	—	—
	ICMP 18697	*Vitis vinifera*	USA	JX010154	JX009987	JX009892	—	JX009557	—	—
	CBS 273.51	*Citrus limon*	Italy	JX010148	JX010054	JX009893	—	JX009558	—	—
	**CBS 953.97***	***Citrus sinensis***	**Italy**	**GQ485605**	**GQ856762**	**JX009894**	**—**	**GQ856782**	**GQ849434**	**—**
	CORCG5	*Vanda* sp.	China	HM034809	HM034807	JX009895	—	HM034801	HM034811	—
	**CBS 112999***	***Citrus sinensis***	**Italy**	**JQ005152**	**JQ005239**	**JX009896**	**JQ005413**	**JQ005500**	**JQ005587**	**—**
C. graminicola	**CBS 130836***	***Zea mays***	**USA**	**JQ005767**	**—**	**JX009897**	**—**	**JQ005830**	**JQ005851**	**—**
C. grevillea	**CBS 132879***	***Grevillea* sp.**	**Italy**	**KC297078**	**KC297010**	**KC296987**	**KC297056**	**KC296941**	**KC297102**	**—**
C. grossum	**CGMCC 3.17614***	***Capsicum* sp.**	**China**	**KP890165**	**KP890159**	**JX009899**	**—**	**KP890141**	**KP890171**	**—**
	CAU31	*Capsicum* sp.	China	KP890166	KP890160	JX009900	—	KP890142	KP890172	—
C. hebeiense	**CGMCC 3.17464***	***Vitis vinifera* cv. Cabernet Sauvignon**	**China**	**KF156863**	**KF377495**	**JX009901**	**—**	**KF377532**	**KF288975**	**—**
C. hederiicola	**MFLU 15-0689***	***Hedera helix***	**Italy**	**MN631384**	**—**	**MN635794**	**—**	**MN635795**	**—**	**—**
C. helleniense	**CPC 26844***	***Poncirus trifoliata***	**Greece, Arta**	**KY856446**	**KY856270**	**KY856186**	**KY856361**	**KY856019**	**KY856528**	**—**
C. henanense	**CGMCC 3.17354**	***C. sinensis*, pathogen**	**China**	**KJ955109**	**—**	**JX009902**	**MZ673835**	**—**	**KJ955257**	**—**
C. hippeastri	**CSSG1***	***Hippeastrum vittatum*, leaf**	**China**	**JQ005231**	**JQ005318**	**JX009903**	**JQ005492**	**JQ005579**	**JQ005665**	**—**
	CBS 241.78	*Hippeastrum* sp.	Netherlands	JX010293	JX009932	JX009904	—	JX009485	—	—
C. horii	**NBRC 7478***	***Diospyros kaki***	**Japan**	**GQ329690**	**GQ329681**	**JX009905**	**—**	**JX009438**	**JX010450**	**—**
C. hymenocallidis	**ICMP 18642***	***Hymenocallis americana***	**China**	**JX010278**	**JX010019**	**JX009906**	**—**	**GQ856775**	**JX010410**	**—**
	**CBS 125378**	***Hymenocallis americana***	**China**	**JX010278**	**JX010019**	**JX009907**	**—**	**GQ856775**	**JX010410**	**—**
C. hystricis	**NBRC 7478***	***Citrus hystrix***	**Italy, Catania**	**KY856450**	**KY856274**	**KY856190**	**KY856365**	**KY856023**	**KY856532**	

Table S2. (cont.)

Species	Accession No[1]	Host	Location	GenBank No[2]						
				ITS	*gadph*	*chs-1*	*his3*	*act1*	*tub2*	*gs*
C. jiangxiense	**CGMCC 3.17363***	***Camellia sinensis*, pathogen**	**China**	**KJ955201**	**KJ954902**	**JX009908**	—	**KJ954471**	**OK236389**	—
C. kahawae	**IMI 319418***	***Coffea arabica***	**Kenya**	**JX010231**	**JX010012**	**JX009912**	**MZ673838**	**JX009452**	**JX010444**	—
C. karstii	CBS 129927	*Anthurium* sp.	Thailand	JQ005206	JQ005293	JX009913	JQ005467	JQ005554	JQ005640	—
	CBS 128524	*Citrullus lanatus*, fruit	New Zealand	JQ005195	JQ005282	JX009914	JQ005456	JQ005543	JQ005629	—
	MAFF 305998	*Cucumis melo*	Japan	JQ005191	JQ005278	JX009915	JQ005452	JQ005539	JQ005625	—
	CBS 110779	*Eucalyptus grandis*	South Africa	JQ005198	JQ005285	JX009916	JQ005459	JQ005546	JQ005632	—
	CBS 127552	*Eugenia uniflora*	Brazil	JQ005217	JQ005304	JX009917	JQ005478	JQ005565	JQ005651	—
	CBS 130235	*Gossypium hirsutum*	Germany	JQ005190	JQ005277	JX009918	JQ005451	JQ005538	JQ005624	—
	CBS 113087	*Malus* sp.	USA	JQ005181	JQ005268	JX009919	JQ005442	JQ005529	JQ005615	—
	CBS 118401	*Pachira* sp., living leaves	China	JQ005192	JQ005279	JX009920	JQ005453	JQ005540	JQ005626	—
	**CGMCC 3.14194***	***Vanda* sp.**	**China**	**HM585409**	**HM585391**	**JX009921**	—	**HM581995**	**HM585428**	—
C. lindemuthianum	CBS 571.97	Phaseolus vulgaris	Brazil	JX546818	JX546722	JX009925	JX546770	JX546626	JX546864	KF178629
	CBS 151.56	Phaseolus vulgaris	France	JX546812	JX546716	JX009926	JX546764	JX546620	JX546858	KF178633
	**CBS 144.31***	**Phaseolus vulgaris**	**Germany**	**JQ005779**	**JX546712**	**JX009927**	**JQ005821**	**JQ005842**	**JQ005863**	**KF178643**
	CBS 152.28	Phaseolus vulgaris	Netherlands	JX546813	JX546717	JX009928	JX546765	JX546621	JX546859	KF178642
	CBS 130841	Phaseolus vulgaris	USA	JX546819	JX546723	JX009929	JX546771	JX546627	JX546865	KF178632
	CBS 523.97	*Phaseolus coccineus*	Costa Rica	JX546815	JX546719	JX009930	—	JX546623	JX546861	—
C. lineola	**CBS 125337***	***Apiaceae***	**Czech Republic**	**GU227829**	**GU228221**	**JX009931**	—	**GU227927**	**GU228123**	—
C. liriopes	**CBS 119444***	***Liriope muscari***	**Mexico**	**GU227804**	**GU228196**	**JX009932**	—	**GU227902**	**GU228098**	—
C. lobatum	**IMI 79736***	***Piper catalpaefolium***	**Trinidad and Tobago**	**MG600768**	**MG600828**	**JX009933**	**MG600912**	**MG600972**	**MG601035**	—
C. magnum	**CBS 519.97***	***Citrullus lanatus***	**USA**	**MG600769**	**MG600829**	**JX009934**	**MG600913**	**MG600973**	**MG601036**	—
	IMI 391662	*Citrullus lanatus*	USA	MG600771	MG600831	JX009935	MG600915	MG600975	MG601038	—
	CBS 575.97	*Citrullus lanatus*	USA	MG600770	MG600830	JX009936	MG600914	MG600974	MG601037	—
	CAUOS2(*)	Chili pepper	China	MZ595839	MZ848400	OK236385	MZ673858	OK236387	MZ67396	—
C. makassarense	**CBS 143664***	***Capsicum annuum***	**Indonesia**	**MH728812**	**MH728820**	**MH805850**	—	**MH781480**	**MH846563**	—
C. malvarum	**CBS 521.97***	**Lavatera trimestris**	**UK**	**KF178480**	**KF178504**	**JX009937**	**KF178553**	**KF178577**	**KF178601**	**KF178626**
C. merremiae	**CBS 124955***	***Merremia umbellata***	**Panama**	**MG600765**	**MG600825**	**JX009938**	**MG600910**	**MG600969**	**MG601032**	—
C. nanhuaensis	**CGMCC 3.18962**	***Ageratina adenophora***	**China**	**OK030870**	**OK513673**	**OK513569**	—	**OK513609**	**OK513639**	—
	YMF1.04990	*Ageratina adenophora*	China	OK030871	OK513674	OK513570	—	OK513610	OK513640	—
C. musae	**CBS 116870***	***Musa* sp.**	**USA**	**JX010146**	**JX010050**	**JX009939**	—	**JX009433**	**HQ596280**	—

Table S2. (cont.)

Species	Accession No[1]	Host	Location	GenBank No[2]						
				ITS	*gadph*	*chs-1*	*his3*	*act1*	*tub2*	*gs*
C. musicola	**CBS 132885***	***Musa* sp.**	**Mexico**	**MG600736**	**MG600798**	**JX009940**	**MG600895**	**MG600942**	**MG601003**	—
C. nigrum	CBS 128507	*Capsicum annuum*	New Zealand	JX546843	JX546747	JX009941	—	JX546651	JX546890	—
	**CBS 169.49***	***Capsicum* sp.**	**Argentina**	**JX546838**	**JX546742**	**JX009942**	—	**JX546646**	**JX546885**	—
C. novae-zelandiae	**ICMP 12944***	***Capsicum annuum*, fruit**	**New Zealand**	**JQ005228**	**JQ005315**	**JX009943**	**JQ005489**	**JQ005576**	**JQ005662**	—
C. nupharicola	**CBS 470.96***	***Nuphar lutea* subsp.**	**USA**	**JX010187**	**JX009972**	**JX009944**	—	**JX009437**	**JX010398**	—
C. nymphaeae	CBS 129928, 216	*Fragaria × ananassa*	USA	JQ948228	JQ948558	JX009945	—	JQ949549	JQ949879	—
	**CBS 515.78***	***Nymphaea alba*, leaf spot**	**Netherlands**	**JQ948197**	**JQ948527**	**JX009946**	—	**JQ949518**	**JQ949848**	—
C. okinawense	MAFF 240517	*Carica papaya*	Japan	MG600767	MG600827	JX009947	—	MG600971	MG601034	—
C. oncidii	**CBS 129828***	***Oncidium* sp., leaf**	**Germany**	**JQ005169**	**JQ005256**	**JX009948**	—	**JQ005517**	**JQ005603**	—
C. orbiculare	CBS 129432	Benincasa hispida	Australia	KF178469	KF178494	JX009949	KF178542	KF178566	KF178590	KF178614
	CBS 133194	Cucumis melo	Japan	KF178459	KF178483	JX009950	KF178532	KF178556	KF178580	KF178604
	**CBS 570.97***	**Cucumis sativus**	**Europe, probably UK**	**KF178466**	**KF178490**	**JX009951**	**KF178539**	**KF178563**	**KF178587**	**KF178603**
	CBS 173.59	Cucumis sativus	Netherlands	KF178463	KF178487	JX009952	KF178536	KF178560	KF178584	KF178608
	CBS 122.24	Cucumis sativus	UK	KF178467	KF178492	JX009953	KF178540	KF178564	KF178588	KF178612
C. orchidearum	**CBS 135131***	***Dendrobium nobile***	**Netherlands**	**MG600738**	**MG600800**	**JX009954**	**MG600897**	**MG600944**	**MG601005**	—
C. panamense	**CBS 125386***	***Merremia umbellata***	**Panama**	**MG600766**	**MG600826**	**JX009955**	**MG600911**	**MG600970**	**MG601033**	—
C. pandanicola	**MFLUCC 17-0571***	***Pandanaceae***	**Thailand**	**MG646967**	**MG646934**	**MG646931**	—	**MG646938**	**MG646926**	—
C. parsonsiae	**ICMP 18590***	***Parsonsia capsularis*, leaf**	**New Zealand**	**JQ005233**	**JQ005320**	**JX009956**	**JQ005494**	**JQ005581**	**JQ005667**	—
C. perseae	**CBS 141365**	***Avocado***	**Israel**	**KX620308**	**KX620242**	**MZ799260**	—	**KX620145**	**KX620341**	—
C. petchii	CBS 118774	*Dracaena sanderana*, living stems	China	JQ005225	JQ005312	JX009957	JQ005486	JQ005573	JQ005659	—
C. phaseolorum 1	CBS 157.36	*Phaseolus radiatus* var. aureus	Japan	GU227896	GU228288	JX009958	—	GU227994	GU228190	—
C. phaseolorum 2	CBS 158.36	*Vigna sinensis*	Japan	GU227897	GU228289	JX009959	—	GU227995	GU228191	—
C. phyllanthi	**MACS 271***	***Phyllanthus acidus***	**India**	**JQ005221**	**JQ005308**	**JX009960**	**JQ005482**	**JQ005569**	**JQ005655**	—
C. piperis	**CPC 21195***	***Piper nigrum***	**Malaysia**	**MG600760**	**MG600820**	**JX009961**	**MG600906**	**MG600964**	**MG601027**	—
C. plurivorum	MAFF 243073	*Amorphophallus rivieri*	Japan	MG600730	MG600793	JX009962	—	MG600936	MG600997	—
	CORCX9	*Arundina graminifolia*	China	HM585398	HM585381	JX009963	—	HM581986	HM585423	—
	**LJTJ30***	***Capsicum annuum***	**China**	**KP748221**	**KP823800**	**JX009964**	—	**KP823741**	**KP823853**	—
	**CBS 125474***	***Coffea* sp.**	**Vietnam**	**MG600718**	**MG600781**	**JX009965**	**MG600887**	**MG600925**	**MG600985**	—
	MAFF 306007	*Lycopersicon*	Japan	MG600728	MG600791	JX009966	—	MG600934	MG600995	—

Table S2. (cont.)

Species	Accession No[1]	Host	Location	GenBank No[2]						
				ITS	*gadph*	*chs-1*	*his3*	*act1*	*tub2*	*gs*
	CMM 3742	*Mangifera indica*	Brazil	KC702980	KC702941	JX009967	—	KC702908	KC992327	—
C. proteae	**CBS 132882***	***Protea* sp.**	**South Africa**	**KC297079**	**KC297009**	**JX009968**	**KC297045**	**KC296940**	**KC297101**	—
	CBS 134301	*Protea* sp.	South Africa	KC842385	KC842379	JX009969	—	KC842373	KC842387	—
C. pseudotheobromicola	**MFLUCC 18—1602***	***Prunus avium***	**China**	**MH817395**	**MH853675**	**MH853678**	—	**MH853681**	**MH853684**	—
C. psidii	**CBS 145.29***	***Psidium* sp.**	**Italy**	**JX010219**	**JX009967**	**JX009970**	—	**JX009515**	**JX010443**	—
C. queenslandicum	**ICMP 1778***	***Carica papaya***	**Australia**	**JX010276**	**JX009934**	**JX009971**	—	**JX009447**	**JX010414**	—
C. rhexiae	**CBS 133134***	***Rhexia virginica***	**USA**	**JX145128**	**MZ664046**	**JX009972**	**MZ673834**	**MZ664127**	**JX145179**	—
C. rusci	**CBS 119206***	***Ruscus* sp.**	**Italy**	**GU227818**	**GU228210**	**JX009973**	—	**GU227916**	**GU228112**	—
C. salsolae	**ICMP 19051***	***Salsola tragus***	**Hungary**	**JX010242**	**JX009916**	**JX009974**	—	**JX009562**	**JX010325**	—
C. siamense	**ICMP 18578***	***Coffea arabica***	**Thailand**	**JX010171**	**JX009924**	**JX009975**	—	**FJ907423**	**JX010404**	—
	CBS 130420	*Jasminum sambac*	Vietnam	HM131511	HM131497	JX009976	—	HM131507	JX010415	—
C. sidae	**CBS 504.97***	**Sida spinosa**	**USA**	**KF178472**	**KF178497**	**JX009977**	**KF178545**	**KF178569**	**KF178593**	**KF178618**
C. simmondsii	CBS130421	*Carica papaya*, fruit	Australia	JQ948276	JQ948606	JX009978	—	JQ949597	JQ949927	—
C. sojae	SAUCC 1407	*Arctium lappa*	China	KT362184	KT362188	JX009979	—	KT362189	KT362185	—
	CAUOS5	*Capsicum* sp.	China	KP890107	KP890138	JX009980	—	—	KP890114	—
	LFN0009	*Glycine max*	Brazil	KT696354	KT696295	JX009981	KT696318	KT696281	KT696288	—
	**ATCC 62257***	***Glycine max***	**USA**	**MG600749**	**MG600810**	**JX009982**	**MG600899**	**MG600954**	**MG601016**	—
C. spinosum	**CBS 515.97***	**Xanthium spinosum**	**Australia**	**KF178474**	**KF178498**	**JX009983**	**KF178547**	**KF178571**	**KF178595**	**KF178620**
C. syzygiicola	**MFLUCC 10-0624***	***Syzygium samarangense***	**Thailand**	**KF242094**	**KF242156**	—	—	**KF157801**	**KF254880**	—
C. tainanense	**CBS 143666***	***Capsicum annuum***	**Taiwan**	**MH728818**	**MH728823**	**MH805845**	—	**MH781475**	**MH846558**	—
C. tebeestii	**CBS 522.97***	**Malva pusilla**	**Canada**	**KF178473**	**KF178505**	**JX009984**	**KF178546**	**KF178570**	**KF178594**	**KF178619**
C. temperatum	**CBS 133122***	***Vaccinium macrocarpon***	**USA**	**JX145159**	**MZ664045**	**JX009985**	**MZ673833**	**MZ664125**	**JX145211**	—
C. theobromicola	**CBS 124945***	***Theobroma cacao***	**Panama**	**JX010294**	**JX010006**	**JX009986**	—	**JX009444**	**JX010447**	—
	CBS 142.31	*Fragaria × ananassa*	USA	JX010286	JX010024	JX009987	—	JX009516	JX010373	—
	MUCL 42294	*Stylosanthes viscosa*	Australia	JX010289	JX009962	JX009988	—	JX009575	JX010380	—
C. ti	**ICMP 4832***	***Cordyline* sp.**	**New Zealand**	**JX010269**	**JX009952**	**JX009989**	—	**JX009520**	**JX010442**	—
C. tofieldiae	CBS 495.85	*Tofieldia calyculata*	Switzerland	GU227801	GU228193	**JX009990**	—	GU227899	GU228095	—
C. torulosum	**ICMP 18586***	***Solanum melongena***	**New Zealand**	**JQ005164**	**JQ005251**	**JX009991**	**JQ005425**	**JQ005512**	**JQ005598**	—
C. trichellum	CBS 217.64	*Hedera helix*	UK	GU227812	GU228204	JX009993	—	GU227910	GU228106	—

Table S2. (cont.)

Species	Accession No[1]	Host	Location	GenBank No[2]						
				ITS	*gadph*	*chs-1*	*his3*	*act1*	*tub2*	*gs*
C. trifolii	**CBS 158.83***	**Trifolium**	**USA**	**KF178478**	**KF178502**	**JX009994**	**KF178551**	**KF178575**	**KF178599**	**KF178624**
C. tropicale	**CBS 124949***	***Theobroma cacao***	**Panama**	**JX010264**	**JX010007**	**JX009995**	**MZ673832**	**JX009489**	**JX010407**	—
C. tropicicola1	**MFLUCC 110114***	***Citrus maxima***	**Thailand**	**JN050240**	**JN050229**	**JX009996**	—	**JN050218**	**JN050246**	—
C. tropicicola2	MFLUCC 100167	*Paphiopedilum bellatulum*	Thailand	JN050241	JN050230	JX009997	—	JN050219	JN050247	—
C. truncatum	**CBS 151.35***	***Phaseolus lunatus***	**USA**	**GU227862**	**GU228254**	**JX009998**	—	**GU227960**	**GU228156**	—
	CBS 120709	*Capsicum frutescens*	India	GU227877	GU228269	JX009999	—	GU227975	GU228171	—
	CBS 345.70	*Glycine max*	Denmark	GU227867	GU228259	JX010000	—	GU227965	GU228161	—
	CBS195.32	*Glycine max*	USA	GU227865	GU228257	JX010001	—	GU227963	GU228159	—
C. verruculosum	IMI 45525	*Crotalaria juncea*	Zimbabwe	GU227806	GU228198	**JX010002**	—	GU227904	GU228100	—
C. viniferum	**GZAAS 5.08601***	***Vitis vinifera*, cv. 'Shuijing'**	**China**	**JN412804**	**JN412798**	**JX010003**	—	**JN412795**	**JN412813**	—
C. walleri	**CBS 125472***	***Coffea* sp., leaf tissue**	**Vietnam**	**JQ948275**	**JQ948605**	**JX010004**	—	**JQ949596**	**JQ949926**	—
C. wuxiense	**CGMCC 3.17894***	***Camellia sinensis***	**China**	**KU251591**	**KU252045**	**JX010005**	—	**KU251672**	**KU252200**	—
C. xanthorrhoeae	**BRIP 45094***	***Xanthorrhoea preissii***	**Australia**	**JX010261**	**JX009927**	**JX010006**	—	**JX009478**	**JX010448**	—
C. yunajiangensis	**GCMCC 3.18964**	***Ageratina adenophora***	**China**	**OK030885**	**OK513686**	**OK513583**		**OK513620**	**OK513649**	—
	YMF1.04997	*Ageratina adenophora*	China	OK030886	OK513687	OK513584		OK513621	OK513650	
C. yunnanense	CBS 132135	*Buxus* sp.	China	JX546804	JX546706	JX010007	—	JX519239	JX519248	—
C. yulongense	**CFCC 50818***	***Vaccinium dunalianum* var. urophyllum**	**China**	**MH751507**	**MK108986**	**MH793605**	—	**MH777394**	**MK108987**	—
Colletotrichum sp.	MAFF 238642	*Dendrobium kingianum*	Japan	JQ005163	JQ005250	JX010008	JQ005424	JQ005511	JQ005597	—
Glomerella cingulata	ICMP 10643	*Camellia × williamsii*	UK	JX010224	JX009908	JX010009	—	JX009540	JX010436	—
Glomerella cingulata var. migrans	CBS 237.49	*Hypericum perforatum*	Germany	JX010238	JX010042	JX009910	—	JX009450	JX010432	—
Glomerella rufomaculans var. vaccini	CBS 124.22	*Vaccinium* sp.	USA	JX010228	JX009950	JX009911	—	JX009536	JX010433	—
Monilochaetes infuscans	**CBS 869.96***	***Ipomoea batatas***	**South Africa**	**JQ005780**	**JX546612**	**JX010010**	—	**JQ005843**	**JQ005864**	—

1. ATCC: American Type Culture Collection, Virginia, USA; BCC: BIOTEC culture collection, Bangkok, Thailand; BRIP: Plant Pathology Herbarium, Department of Employment, Economic, Development and Innovation, Queensland, Australia; CBS: Culture collection of the Centraalbureau voor Schimmelcultures, Fungal Biodiversity Centre, Utrecht, The Netherlands; CGMCC: China General Microbiological Culture Collection Center, Beijing, China; CMM: Culture Collection of Phytopathogenic Fungi Prof. Maria Menezes, Federal Rural University of Pernambuco, Brazil; CPC: Culture collection of Pedro Crous, housed at CBS; COAD: Coleção Octávio Almeida Drummond, Viçosa, Brazil; GZAAS: Guizhou Academy of Agricultural Sciences, Guiyang, China; IMI: Culture collection of CABI Europe UK Centre, Egham, UK; MAFF: MAFF Genebank Project, Ministry of Agriculture, Forestry and Fisheries, Tsukuba, Japan; MFLUCC: Culture collection of the Mae Fah Luang University, Chiang Rai, Thailand ; SAUCC: Culture collection of the Department of Plant Pathology, College of Plant Protection,Shenyang Agricultural University, China;
2. ITS: internal transcribed spacers and intervening 5.8S nrDNA; GAPDH: partial glyceraldehyde–3–phosphate dehydrogenase gene; CHS–1: partial chitin synthase–1 gene; HIS: partial histone H3 gene; ACT: partial actin gene; TUB2: partial beta–tubulin gene; GS: intron 2 glutamine synthetase.

* = ex–type or authentic culture, (*) = ex–type or authentic culture of synonymised taxon. Strains studied in this paper are in bold font.
