# Supplementary material for: Colletotrichum Species Associated with Anthracnose Disease of Watermelon (Citrullus lanatus) in China
Source: J Fungi (Basel). 2022 Jul 28;8(8):790. doi: 10.3390/jof8080790 (PMC9410023; doi:10.3390/jof8080790)
Supplement: Supplementary file 1 [file jof-08-00790-s001.zip › Table s3.pdf]

Table S3. The sizes of seta, conidia, appresoria and ascospores of the representative isolates of *Colletotrichum* spp. obtained in this study.

Species	Strain number	Seta on SNA	Conidia on PDA			Appresoria on PDA			Ascospores on OA		
		Length (μm) × Width (μm)[1]	Length (μm) × Width (μm)[1]	Means ± SD of conidia sizez[2]	L/W ratio	Length (μm) × Width (μm)[1]	Means ± SD of conidia sizez[2]	L/W ratio	Length (μm) × Width (μm)[1]	Means ± SD of conidia sizez[2]	L/W ratio
C. aenigma	CAASZB1	41.8–80.9 × 3.9–6.5	13.4–18.2 × 4.4–6.3	15.6± 1.1 × 5.5± 0.4	2.9	8.4–12.7 × 6.4–10.8	10.8 ± 1.1 × 8.3 ± 1.1	1.3	14.8–18.7 × 4.8–7.4	16.4 ± 0.9 × 5.8 ± 0.6	2.8
C. chlorophyti	CAASZG32	47.2–141.3 × 3.9–6.2	15.9–20.6 × 5.4–6.4	17.5 ± 1.2 × 5.2 ± 0.5	3.4	9.2–16.1 × 5.9–12.5	11.8 ± 1.6 × 8.9 ± 1.4	1.3	/	/	/
C. citrulli	CAASZT52	42.1–78.9 × 3.1–6.4	14.8–18.3 × 4.8–6.5	16.4± 0.9 × 5.5± 0.5	3	7.4–15.1 × 5.0–10.0	10.8 ± 1.7 × 7.6 ± 1.2	1.4	/	/	/
C. fructicola	CAASZA2	40.8–85.2 × 3.2–5.5	12.0–16.8 × 3.8–7.0	13.6± 1.1 × 4.9± 0.8	2.8	7.3–13.4 × 5.3–11.4	10.4 ± 1.5 × 8.0± 1.2	1.3	16.6–21.5 × 4.2–6.0	19.3 ± 1.7 × 4.7± 0.4	4.2
	CAASZN18	/	/	/	/	/	/	/	16.6–22.2 × 3.8–6.1	18.8± 1.5 × 5.0± 0.6	3.8
C. jiangxiense	CAASZN28	/	14.1–22.1 × 4.2–8.2	17.4 ± 2.0 × 5.2± 0.8	3.4	8.0–13.3 × 6.1–10.0	10.5 ± 1.2 × 7.8 ± 0.9	1.4	/	/	/
	CAASZN20	/	10.0–20.0 × 4.2–6.3	13.3 ± 2.8 × 5.1± 0.5	2.6	7.0–11.4 × 6.4–9.1	9.2 ± 1.0 × 7.6± 0.8	1.2	/	/	/
C. kaifengense	CAASZK33	82.4–177.3 × 4.4–6.9	13.3–19.8 × 4.0–6.4	17.4 ± 1.7 × 4.9 ± 0.5	3.5	8.3–14.5 × 5.4–11.2	11.1 ± 1.9 × 7.9 ± 1.4	1.5	/	/	/
C. karstii	CAASZG29	39.6–73.7 × 3.6–7.1	13.3–15.9 × 5.1–6.9	14.0± 1.9 × 6.2± 0.4	2.3	9.1–14.3 × 6.6–10.8	10.6 ± 1.2 × 8.7 ± 1.1	1.2	11.5–16.6 × 5.6–7.7	14.0 ± 1.9 × 6.2 ± 0.4	2.3
	CAASZY9	/	12.5–17.5 × 4.9–7.0	14.9 ± 1.3 × 5.8 ± 0.5	2.6	7.6–13.0 × 5.9–10.4	9.6 ± 1.0 × 7.8 ± 0.8	1.3	/	/	/
	CAASZY10	33.0–66.5 × 2.9–5.6	12.3–17.1 × 5.2–8.2	14.7 ± 0.9 × 6.5 ± 0.7	2.3	8.4–13.1 × 6.4–9.1	9.6 ± 1.1 × 7.6 ± 0.7	1.3	/	/	/
	CAASZY14	42.8–63.0 × 3.6–7.3	13.6–16.6 × 6.1–7.5	15.1 ± 0.8 × 6.6 ± 0.3	2.3	5.9–11.1 × 4.8–9.1	8.4 ± 1.3 × 7.2 ± 1.1	1.3	/	/	/
C. magnum	CAASZF5	84.7–126.6 × 3.9–6.5	14.8-19.1 × 4.7-7.5	17.3 ± 1.2 × 6.3 ± 0.6	2.9	7.0–13.4 × 6.0–11.3	10.8 ± 1.4 × 6.0 ± 1.2	1.4	/	/	/
	CAASZK1	85.3-163.4 × 3.6-5.9	13.7-19.8 × 4.3-6.5	16.9 ± 1.5 × 5.4 ± 0.5	3.2	6.7–16.1 × 8.4–16.6	13.5 ± 2.5 × 10.7 ± 2.0	1.3	/	/	/
	CAASZK3	109.3–220.6 × 3.9–5.6	17.6–21.5 × 4.9–6.3	19.0 ± 1.0 × 5.4 ± 0.5	3.6	8.6–15.8 × 5.1–10.9	12.6 ± 1.8 × 7.7 ± 0.9	1.7	/	/	/
	CAASZK34	97.5–195.0 × 3.3–5.9	13.8–24.1 × 4.4–6.5	18.3 ± 2.4× 5.5 ± 0.5	3.3	8.4–15.9 × 4.9–11.4	11.8 ± 1.8 × 4.9 ± 11.4	1.5	/	/	/
C. nigrum	CAASZX53	/	10.9–16.7 × 3.5–6.5	13.6 ± 1.7 × 4.5 ± 0.6	3.1	8.8–17.8 × 5.7–8.5	12.8 ± 2.1 × 7.0 ± 0.9	1.8	/	/	/
	CAASZX80	/	9.7–17.7 × 3.1–4.8	11.7 ± 1.1× 4.0 ± 0.4	2.9	9.5–18.7 × 6.2–8.3	13.1 ± 2.2 × 7.4 ± 0.6	1.8	/	/	/
C. nymphaeae	CAASZN14	/	10.5–15.8 × 3.1–6.3	13.3 ± 1.5 × 4.2 ± 0.7	3.2	7.8–12.4 × 5.4–9.4	9.7 ± 1.2 × 7.1 ± 1.0	1.4	/	/	/
	CAASZN52	/	11.5–17.9 × 3.7–6.1	14.1 ± 1.4 × 4.8 ± 0.5	3.0	7.1–10.5 × 5.7–8.0	8.9 ± 0.9 × 6.9 ± 0.7	1.3	/	/	/
C. orbiculare	CAASZN2	39.6–73.74 × 3.9–6.0	10.0–13.0 × 3.1–6.1	11.3 ± 0.7 × 5.2 ± 0.6	2.2	8.1–13.9 × 5.2–8.5	10.5 ± 1.3 × 6.7 ± 1.0	1.6	/	/	/
	CAASZJ2	35.1–56.0 × 3.7–5.4	10.0–15.8 × 5.2–6.9	12.6 ± 1.3 × 6.1 ± 0.5	2.2	7.8–10.9 × 5.3–6.9	9.3 ± 1.0 × 6.0 ± 0.5	1.6	/	/	/
	CAASZT46	/	9.5–12.5 × 5.3–6.6	11.2 ± 0.8 × 5.9 ± 0.3	1.9	8.7–16.3 × 5.3–7.7	10.4 ± 1.8 × 6.5 ± 0.6	1.6	/	/	/
	CAASZK46	36.4–59.8 × 3.4–6.1	10.0–15.5 × 4.6–6.9	12.7 ± 1.4 × 5.4 ± 0.6	2.4	6.4–11.4 × 4.4–7.8	8.9 ± 1.1 × 5.8 ± 0.8	1.0	/	/	/

Table S3. (cont.)

Species	Strain number	Seta on SNA	Conidia on PDA			Appresoria on PDA			Ascospores on OA		
		Length (μm) × Width (μm)[1]	Length (μm) × Width (μm)[1]	Means ± SD of conidia sizez[2]	L/W ratio	Length (μm) × Width (μm)[1]	Means ± SD of conidia sizez[2]	L/W ratio	Length (μm) × Width (μm)[1]	Means ± SD of conidia sizez[2]	L/W ratio
	CAASZA9	/	10.7–18.3 × 4.1–6.1	13.7 ± 1.9 × 4.9 ± 0.6	2.8	8.2–14.8 × 5.9–10.8	11.5 ± 1.4 × 8.5 ± 1.1	1.4	13.5–20.1 × 5.5–7.5	17.5 ± 1.6 × 6.5 ± 0.5	3.2
	CAASZA30	/	12.4–16.5 × 5.6–6.4	14.6 ± 1.1 × 6.2 ± 0.6	2.4	8.3–14.5 × 6.0–8.9	12.6 ± 1.6 × 7.5 ± 0.6	1.7	/	/	/
C. qilinense	CAASZK13	82.7–203.3 × 3.5–6.8	17.5–23.2 × 4.7–6.0	19.7 ± 1.4× 5.4 ± 0.4	3.7	8.1–15.8 × 5.1–10.8	10.6 ± 2.1 × 7.7± 1.5	1.4	/	/	/
C. sojae	CAASZM39	/	13.1–18.7 × 4.6–7.1	15.9 ± 1.5 × 6.0 ± 0.5	2.7	8.2–16.7 × 7.1–11.9	12.9 ± 2.2 × 7.1 ± 11.9	1.4	/	/	/
	CAASZM32	70.2–158.4 × 3.9–6.1	12.7–19.1 × 4.7–6.6	16.3 ± 1.5 × 5.6 ± 0.5	2.8	8.5–14.0 × 6.3–9.2	10.4 ± 1.3 × 7.6 ± 0.7	1.4	/	/	/
C. truncatum	CAASZX14	65.0–187.4 × 3.7–7.5	27.0–33.1 × 3.3–5.4	30.6 ± 1.7 × 4.3 ± 0.5	6.5	6.5–10.5× 4.6–8.7	9.1 ± 0.9 × 6.3 ± 0.8	1.5	/	/	/
	CAASZG10	77.9–140.6 × 5.3–8.7	23.2–29.8 × 3.3–4.7	26.1 ± 1.6 × 3.8 ± 0.4	7.0	8.8–15.5 × 5.6–9.4	11.9 ± 1.7 × 7.4 ± 0.8	1.6	/	/	/
	CAASZG41	67.3–236.9 × 4.8–7.1	18.8–28.0 × 3.3–5.6	24.8 ± 2.1 × 3.3 ± 5.6	5.7	8.0–17.6 × 4.9–9.7	11.4 ± 2.1× 7.4 ± 1.1	1.6	/	/	/

1 Numbers indicate minimum and maximum sizes for length or width of 30 seta, conidia, and appresoria recorded from the representative strains of Colletotrichum spp. obtained in this study.

2 Significance at P = 0.05 level. Numbers indicate mean conidia, appresoria sizes of each representative strain calculated by the statistical analysis. Data were analyzed with SPSS Statistics 19.0 (WinWrap® Basic; http://www.winwrap.com) by one-way ANOVA, and means were compared using Duncan’s test at a significance level of P = 0.05. SD: standard deviation.
